# Lipid Droplets in Unicellular Photosynthetic Stramenopiles

**DOI:** 10.3389/fpls.2021.639276

**Published:** 2021-04-22

**Authors:** Nolwenn Guéguen, Damien Le Moigne, Alberto Amato, Juliette Salvaing, Eric Maréchal

**Affiliations:** Laboratoire de Physiologie Cellulaire et Végétale, INRAE, CNRS, CEA, IRIG, CEA Grenoble, Université Grenoble Alpes, Grenoble, France

**Keywords:** heterokont, stramenopile, *Nannochloropsis*, *Microchloropsis*, *Phaeodactylum*, lipid droplet (LD), triacylglycerol (TAG), seipin

## Abstract

The Heterokonta or Stramenopile phylum comprises clades of unicellular photosynthetic species, which are promising for a broad range of biotechnological applications, based on their capacity to capture atmospheric CO_2_ via photosynthesis and produce biomolecules of interest. These molecules include triacylglycerol (TAG) loaded inside specific cytosolic bodies, called the lipid droplets (LDs). Understanding TAG production and LD biogenesis and function in photosynthetic stramenopiles is therefore essential, and is mostly based on the study of a few emerging models, such as the pennate diatom *Phaeodactylum tricornutum* and eustigmatophytes, such as *Nannochloropsis* and *Microchloropsis* species. The biogenesis of cytosolic LD usually occurs at the level of the endoplasmic reticulum. However, stramenopile cells contain a complex plastid deriving from a secondary endosymbiosis, limited by four membranes, the outermost one being connected to the endomembrane system. Recent cell imaging and proteomic studies suggest that at least some cytosolic LDs might be associated to the surface of the complex plastid, via still uncharacterized contact sites. The carbon length and number of double bonds of the acyl groups contained in the TAG molecules depend on their origin. *De novo* synthesis produces long-chain saturated or monounsaturated fatty acids (SFA, MUFA), whereas subsequent maturation processes lead to very long-chain polyunsaturated FA (VLC-PUFA). TAG composition in SFA, MUFA, and VLC-PUFA reflects therefore the metabolic context that gave rise to the formation of the LD, either via an early partitioning of carbon following FA *de novo* synthesis and/or a recycling of FA from membrane lipids, e.g., plastid galactolipids or endomembrane phosphor- or betaine lipids. In this review, we address the relationship between cytosolic LDs and the complex membrane compartmentalization within stramenopile cells, the metabolic routes leading to TAG accumulation, and the physiological conditions that trigger LD production, in response to various environmental factors.

## Introduction

Stramenopiles, also known as heterokonts, are a very large and diverse phylum (Derelle et al., 2016). They are originally defined as eukaryotic protists producing asymmetrically biflagellated zoospores, characterized by an anterior flagellum bearing ciliary hairs and a short posterior one (Cavalier-Smith, 2018). They include more than 100,000 species ranging from microscopic unicellular organisms to large multicellular organisms such as the giant seaweeds of the kelp forest. Most of the groups belonging to this phylum are photosynthetic, but it also includes some colorless non-photosynthetic stramenopiles such as Thraustochytrids populating a variety of habitats from polar areas to tropical mangroves (Fossier Marchan et al., 2018; Strassert et al., 2019).

**FIGURE 1:**
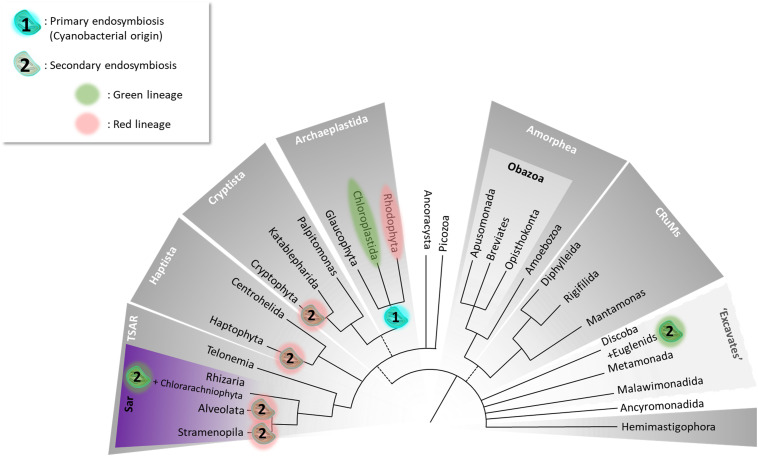
The Tree of Eukaryotes. The colored groupings correspond to currently defined “supergroups.” Unresolved branching orders among lineages are shown as multifurcations. Broken lines reflect minor uncertainties about the monophyly of certain groups. (1) Unique primary endosymbiosis event; (2) multiple secondary endosymbiosis events identified by Oborník (2019) as “complex endosymbioses.” The tree topology was adapted from Burki et al. (2020).

Efforts to propose a consensus eukaryotic Tree of Life (eToL) have benefited from the progress in phylogenomics developed in the past decades and the recent addition of evolutionarily key protist taxa. In the latest eToL, based almost entirely on multigene molecular phylogenies (Figure 1; Burki et al., 2020), microalgae cluster in numerous supergroups. Previously, all organisms bearing a red algal-derived plastid were assigned to Chromalveolata following the assumption that their plastids had been acquired from a common ancestor. Nowadays, this hypothesis is controversial, with a reassignment of Chromalveolata lines into TSAR (telonemids, stramenopiles, alveolates, and Rhizaria), Haptista, and Cryptista. Stramenopiles belong to the TSAR supergroup.

Oleaginous stramenopile species, such as the diatom *Phaeodactylum tricornutum* and the eustigmatophyte *Nannochloropsis s.l.* (*sensu lato*, i.e., including the recently described genus *Microchloropsis*, Fawley et al., 2015) have attracted the attention for a broad range of potential biotechnological applications. Oleaginous strains accumulate oil rich in triacylglycerol (TAG), whose fatty acids (FAs) comprise long-chain saturated or monounsaturated FAs (SFA, MUFA, with 16 or 18 carbons), and very long-chain polyunsaturated FA (VLC-PUFA, with 20 or 22 carbons and up to five or six double bonds). TAGs enriched in SFA and MUFA are often considered as a feedstock for biofuels and green chemistry (Lupette and Maréchal, 2018), whereas TAGs enriched in VLC-PUFA are valuable for feed, food, and human health (Lupette and Benning, 2020). Understanding the physiological contexts leading to the formation of TAG with various FA composition is therefore essential.

In this review, we summarize the current knowledge on the relationship between cytosolic lipid droplets (LDs) and the complex membrane compartmentalization within stramenopile cells, the metabolic routes leading to TAG accumulation, and the physiological conditions in which LDs are produced, in response to various environmental factors.

## Evolution and Plastid Architecture in Stramenopiles

### The Origin of Secondary Plastids in Stramenopiles

**FIGURE 2:**
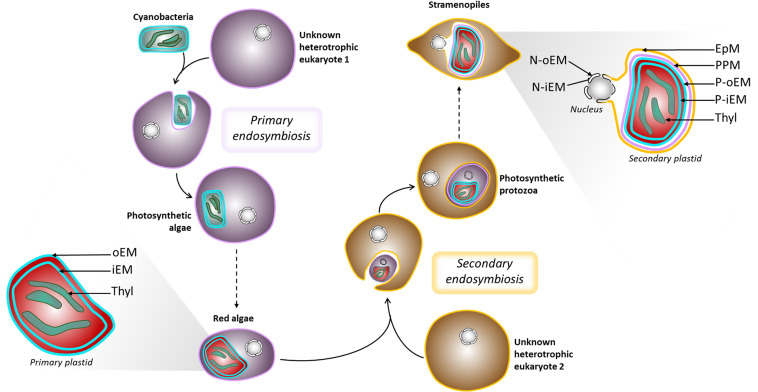
Schematic representation of plastid evolution. Schematic representation of primary and secondary endosymbiosis, and organelle architecture. EpM, epiplastidial membrane; PPM, periplastidial membrane; P-oEM and P-iEM, plastid outer and inner envelope membranes respectively; Thyl, thylakoids; N-oEM and P-iEM, nucleus outer and inner envelope membranes respectively.

Eukaryotes acquired photosynthesis through the engulfment of a photosynthetic cyanobacterium (primary endosymbiosis) or of another photosynthetic eukaryote (secondary endosymbiosis). The engulfed organisms underwent genetic reductions, a loss of autonomy to such an extent that they fully integrated the host cell, giving rise to organelles, usually photosynthetic, collectively termed “plastids.” The initial acquisition of a cyanobacterium by an unknown ancestral heterotrophic eukaryote occurred around 1 to 1.5 billion years ago (Jensen and Leister, 2014) leading to the emergence of the “primary plastid”—the so-called chloroplast (Figure 2). This view is now considered as partly incomplete, since numerous chloroplastic proteins, including the majority of enzymes involved in membrane lipid biosynthesis, are of non-cyanobacterial origin (Petroutsos et al., 2014; Cenci et al., 2017; Sato and Awai, 2017; Marechal, 2018). It is thus considered that other prokaryotes were present with the ancestral cyanobacterium and contributed to the settlement and integration of the structure that eventually became the primary chloroplast, as we know it today (Cenci et al., 2017). Unicellular organisms possessing a primary plastid all derive from the same first endosymbiosis event. They are called Archaeplastida (Adl et al., 2005) and radiated into three major lineages, the Glaucophyta, the Rhodophyceae or red algae, and the Chloroplastida (Adl et al., 2005), also known as Viridiplantae (Cavalier-Smith, 1981). Recently, Li et al. (2020) described the new phylum/division Prasinodermophyta and demonstrated that Prasinodermophyta belongs to Viridiplantae (Li et al., 2020).

Secondary endosymbiosis refers to the acquisition of a secondary plastid by eukaryote-to-eukaryote endosymbiosis (Dorell et al., 2017). The engulfed organism is indeed a photosynthetic eukaryote containing a primary plastid (Figure 2). Secondary endosymbiosis is thought to have occurred several times during evolution and has been performed by heterotroph eukaryotes, consequently becoming phototrophs (Reyes-Prieto et al., 2007). Higher-order endosymbiosis events also took place during evolution and involved more complex interactions, also leading to complex plastid formation (Keeling, 2010, 2013; Jackson et al., 2012; Oborník, 2019).

*Nannochloropsis s.l.* and *P. tricornutum* both stem from two endosymbiosis events, where the last endosymbiosis involved the engulfment of a microalga from the red lineage. However, the second endosymbiosis may imply different red algae and/or different heterotrophic cells (Keeling, 2013), as well as distinct patterns of horizontal gene transfers (Fan et al., 2020; Vancaester et al., 2020). Thus, although phylogenetically close, their metabolisms might differ.

### The Complex Plastids of *Phaeodactylum* and *Nannochloropsis s.l.* Present a Nucleus-Plastid Continuum

A common feature of complex plastids is the presence of more than two surrounding membranes. Some complex plastids can even still contain a relic of the nucleus from the ancestral eukaryotic endosymbiont, called the nucleomorph (Maier et al., 1991; Gilson et al., 2006; Curtis et al., 2012). *Nannochloropsis s.l.* and *P. tricornutum* possess plastids bounded by four membranes and lack the nucleomorph. The lipid composition of plastidial membranes is still unknown and is often inferred based on their evolutionary origins.

The origin of the two innermost membranes of the plastid is not or little debated, and assumed to correspond to the chloroplast envelope of the symbiont, called the outer and inner envelope membranes (P-oEM and P-iEM, respectively). However, different suppositions have been made about the origin of the additional bounding membranes. The outermost membrane, called the epiplastidial membrane (EpM), could derive from the host phagocytic membrane. Underneath, the periplastidial membrane (PPM) is considered to derive from the symbiont plasma membrane (Grosche et al., 2014). Alternatively, it has been hypothesized that the outer envelope membranes of red complex plastids could derive from the host ER (Gould et al., 2015). Interestingly, a vesicular network forming a “blob-like” structure has been detected between the PPM and the EpM in *P. tricornutum*, but its function is still elusive (Kilian and Kroth, 2004; Flori et al., 2016).

The EpM can also be found under the name “chloroplastic endoplasmic reticulum,” as its particularity in *P. tricornutum* and *Nannochloropsis s.l.* is to be continuous with the ER and the outer membrane of the nuclear envelope, forming a nucleus-plastid Continuum (NPC) (Murakami and Hashimoto, 2009; Flori et al., 2016). In addition, membrane contact sites were detected between the inner membrane of the nuclear envelope and the PPM (Flori et al., 2016). Such a tight association between the plastid and the nucleus is hypothesized to facilitate exchanges of small molecules, RNAs, proteins, etc.

Based on the suggested origin of the four membranes around the secondary plastid, it is plausible but still not demonstrated that the two innermost membranes contain plastid-specific lipids such as monogalactosyldiacylglycerol, digalactosyldiacylglycerol, and sulfoquinovosyldiacylglycerol (MGDG, DGDG, and SQDG, respectively) (Boudière et al., 2014), whereas the two outermost membranes may be related to the ER lipid composition, with lipids such as phosphatidylcholine (PC) and betaine lipids (Boudière et al., 2014; Dolch and Maréchal, 2015).

The events that led to the emergence of secondary plastids are critical milestones in the evolution of eukaryotes, and their impact on cell biology is still poorly understood. The structure of the complex/secondary plastids differs radically from other well-known semi-autonomous organelles such as the mitochondria or “classical” chloroplasts. The tight structural and physical interactions of the secondary plastid with other organelles of the endomembrane system, such as the ER, the nucleus, or uncharacterized networks of vesicles, challenge our understanding of cellular processes usually assumed to be restricted to the ER. This holds particularly true for the formation of LDs in oleaginous stramenopiles. Such missing knowledge is critical, when trying to optimize stramenopile algae for biotechnological purposes.

## Composition of the Lipid Droplet, a Storage for Triacylglycerol and Other Lipophilic Compounds

### A Limiting Monolayer Made of Glyco-, Phospho-, and Betaine Lipids

While cytosolic LDs are classically described to be limited by a phospholipid monolayer in non-photosynthetic eukaryotes (Tauchi-Sato et al., 2002; Bartz et al., 2007), this is not always the case for some photosynthetic microalgae recently characterized, from Chlorophytes to Stramenopila (Peled et al., 2011; Tsai et al., 2015; Lupette et al., 2019). Glycerolipid composition of LDs in *P. tricornutum* has been described on a fraction purified with great attention (Lupette et al., 2019). Besides the 99.0 mol% of TAG, it comprises a betaine lipid, diacylglycerylhydroxymethyl-*N*,*N*,*N*-trimethyl-beta-alanine or DGTA (0.4 mol%), a plastid sulfolipid SQDG (0.35 mol%), and a prominent phospholipid of the ER, PC (0.15 mol%). The latter three lipids probably make up the LD bounding monolayer. Brassicasterol is also copurified with LDs, presumed to be included in the limiting monolayer, where it represents almost 5 mol% of total sterols and glycerolipids (Lupette et al., 2019). Such a profile differs from the general assumption of LD monolayer exclusively composed of phospholipids. A set of possible hypotheses about the site of biogenesis of lipid droplets is discussed further below.

To our knowledge, lipid composition of the LD membrane in *Nannochloropsis s.l.* has yet to be described, although it has been referred to as a phospholipid monolayer (Vieler et al., 2012a). In the light of recent studies in microalgae, it appears of pivotal importance to verify lipid composition in *Nannochloropsis s.l.* LDs as well (Peled et al., 2011; Tsai et al., 2015; Lupette et al., 2019).

Proteins are inserted into or associated to the LD bounding lipid monolayer. Their nature and function are discussed further, later in this review.

### The Core of the Lipid Droplet: More Than Triacylglycerol Molecules

The LD core is mainly made of TAGs, but pigments have also been detected, and the presence of other hydrophobic compounds cannot be excluded. Cytological studies very often highlight the detection of pigmented LDs in the cytosol in a large variety of species, like in the green alga *Haematococcus lacustris* (as *H. pluvialis* in Peled et al., 2011; Gao et al., 2013). LDs in both *P. tricornutum* and *Nannochloropsis s.l.* also contain carotenoids (Vieler et al., 2012a; Yoneda et al., 2016; Lupette et al., 2019). Pigment detection has to be taken cautiously given some plastoglobules from the stroma of plastids may be at the origin of part of the carotenoids unavoidably contaminating cytosolic LD fractions. In *P. tricornutum*, fucoxanthin, and β-carotene are the main carotenoids in LD-enriched fractions, while only traces of other pigments were detected (Lupette et al., 2019). The light harvesting complex in *P. tricornutum* is made of fucoxanthin, chlorophyll a, and chlorophyll c. The presence of fucoxanthin and absence of chlorophylls in purified LDs suggest that in *P. tricornutum* LD, fucoxanthin may be part of a distinct pool (Lupette et al., 2019). The function of LD pigments is currently poorly understood in stramenopiles and often considered as secondary, compared with the function of LDs as TAG reserves.

## Ld Formation, a Cell Response to Adapt to a Challenging Environment

### Growth Conditions and Abiotic Parameters of the Environment Influence Lipid Droplet Formation

Several abiotic factors influence TAG accumulation in microalgae, though it is often difficult to identify which specific parameter, or combination of parameters, indeed triggers this accumulation. In standard “unstressed” conditions, light regime and intensity play a crucial role on lipid metabolism (Janssen et al., 2018, 2019b). Basically, TAG often accumulates during the light period and is consumed in the dark (Sukenik and Carmeli, 1990; Fábregas et al., 2002; Chauton et al., 2013; Poliner et al., 2015). Temperature variations were also reported to alter the extent of TAG accumulation (Pasquet et al., 2014; Gill et al., 2018). When light or temperature values exceed the tolerance threshold, becoming hence a stressing factor, TAG accumulation may be triggered (Alboresi et al., 2016). The availability of an organic carbon source can also increase the magnitude of TAG production in unstressed conditions. *P. tricornutum* and *Nannochloropsis s.l.* can grow in mixotrophy, with the provision of additional carbon sources like glycerol, glucose, starch, arginine, and acetate, each with a distinct effect on TAG accumulation (Cerón Garcí et al., 2000; Fang et al., 2004; Villanova et al., 2017; Menegol et al., 2019; Munz et al., 2020).

Various environmental stresses can affect microalgal physiology and lead to the production of LDs in the cytosol. Nutrient starvation is widely studied (Vieler et al., 2012b; Simionato et al., 2013; Mayers et al., 2014; Abida et al., 2015; Mühlroth et al., 2017; Liang et al., 2019a,b), nitrogen in particular (Hu et al., 2008). Phosphate and nitrate limitations are supposed to induce an imbalance between carbon and the missing nutrient, with a carbon excess diverted to storage forms including TAG. Cells exposed to nitric oxide, hydrogen peroxide, and other oxidative reactive species, also respond by increasing TAG accumulation in LD (Dolch et al., 2017a; Conte et al., 2018). A plethora of chemicals proved efficient in triggering TAG accumulation in *P. tricornutum* (Conte et al., 2018) and *Nannochloropsis s.l.* (Franz et al., 2013).

### Triacylglycerol Formation, From *de novo* Synthesis to Lipid Remodeling

Nitrogen starvation can trigger a rapid TAG accumulation in organisms spanning from bacteria (Santucci et al., 2019), green algae (Goncalves et al., 2016), dinoflagellates (Weng et al., 2015), to different stramenopiles (Li et al., 2014; Abida et al., 2015; Jia et al., 2015; Dellero et al., 2018b; Janssen et al., 2019a). The mechanisms of TAG synthesis are well described, and two routes lead to the accumulation of neutral lipids. Part of it stems from *de novo* biosynthesis, the rest from intense lipid remodeling.

Triacylglycerol molecules are composed of three FAs esterified onto a glycerol backbone. The Kennedy pathway that leads to TAG *de novo* formation, also called the acyl-CoA-dependent pathway, is partially involved in the synthesis of all other glycerolipids. The series of reactions for this pathway can occur in both the ER, using acyl-CoA (canonical Kennedy pathway), and the plastid, using acyl-ACP as substrate (Kennedy-like pathway) (Kennedy and Weiss, 1956). The last step of TAG formation can alternatively occur using FA transferred from an existing membrane glycerolipid used as a donor. This pathway is then termed acyl-CoA independent (Dahlqvist et al., 2000).

#### Common Initial Steps for the Formation of Membrane Glycerolipids and Triacylglycerol

The scaffolding starts with the esterification of an acyl-CoA at position *sn-*1 of a glycerol-3-phosphate (G3P) by a glycerol-3-phosphate-*sn*1-acyl-CoA-acyltransferase (GPAT) leading to lysophosphatidic acid (LPA). The addition of a second acyl-group at position sn-2 by a lysophosphatidic acid acyltransferase (LPAT) produces phosphatidic acid (PA). PA can be used either to synthesize phosphatidylglycerol (PG) via a cytidine diphosphate diacylglycerol intermediate or dephosphorylated by a PA phosphatase (PAP) to produce diacylglycerol (DAG). Most glycerolipids can be synthesized from DAG. In the plastid, DAG is the substrate for SQDG and MGDG syntheses, while in the ER, DAG is the substrate for the synthesis of PC, phosphatidylethanolamine (PE), and betaine lipids. The last step of TAG synthesis is commonly performed in the ER by a DAG acyltransferase (DGAT) that esterifies a third acyl-CoA in position *sn-3*. DAG used in the last step of the Kennedy pathway can stem from *de novo* synthesis as described here, but also from the recycling of membrane lipids (Li-Beisson et al., 2010, 2019; Boudière et al., 2012; Petroutsos et al., 2014).

Schematically, FAs *de novo* synthesized in the plastid of *P. tricornutum* and *Nannochloropsis s.l.* contain 16 carbons, whereas longer chained FAs are produced by an elongation process occurring in the ER (Dolch et al., 2017b). The early steps of the Kennedy-like pathway in the plastid is often referred to as the prokaryotic pathway, although this term was recently contested (Sato and Awai, 2017), and membrane glycerolipids characterized by 16-carbon FAs at position *sn*-2 are considered to harbor a prokaryotic signature. Elongation of FAs to 18 or more carbons involves the export of C16 from the plastid to the ER. The ER pathway is often referred to as the eukaryotic pathway, and leads to glycerolipids with various signatures, depending on the species. While such a distinction between topologically distinct pathways is quite evident in plants (Roughan and Slack, 1982), in most stramenopiles, a more thorough investigation is needed to depict the exact origin of the different building blocks of glycerolipids (Mühlroth et al., 2013).

In *Nannochloropsis s.l.* and *P. tricornutum*, both prokaryotic (plastid) and eukaryotic (ER) pathways seem to provide DAG substrates for TAG biosynthesis (Radakovits et al., 2012; Vieler et al., 2012b). A recent analysis in *N. oceanica* has focused on the four copies of LPATs, addressing their subcellular location and function in the synthesis of eukaryotic precursors, based on single and double knockout (KO) studies (Nobusawa et al., 2017). NoLPAT1 proved to be mainly involved in the transfer of 16:1 to the *sn*-2 position of LPA used for the synthesis of membrane glycerolipids, particularly PC and DGTS. This isoform does not influence TAG biosynthesis. By contrast, NoLPAT4 transfers 16:0 to the *sn*-2 position of LPA purely dedicated to TAG. Eventually, NoLPAT2 and NoLPAT3 are mainly involved in the transfer of 18:1 at the *sn-*2 position of precursors used for PC, PE, and of 16:0 at the *sn*-2 position of precursors for TAG (Nobusawa et al., 2017). Thus, in the early step of the Kennedy pathway, LPAT isoforms seem to control the fate of the produced phosphatidic acid, upstream membrane glycerolipid, and/or TAG pathways. Based on GFP-fusion analyses, NoLPAT1 and NoLPAT2 were likely localized at the ER, whereas NoLPAT3 and NoLPAT4 were located at the periphery of cytosolic LDs (Nobusawa et al., 2017). Based on this differential pattern, the role of NoLPAT2 in TAG formation would suggest that membranes related to the ER may be a platform for LD formation, at least in the early stages of LD biogenesis. The location of NoLPAT3 and NoLPAT4 suggests that they may be involved in the production of TAG loaded in more mature LDs. The location of NoLPAT3 further suggests that some part of the membrane lipid synthesis might occur at the vicinity of LDs. The role of LPATs is therefore likely to be critical in the control of metabolic routes directed to membrane glycerolipids, TAG, or both.

In addition, DAG may derive from membrane glycerolipids, like PC, via the action of phospholipases C (PLC) or phosphatidylcholine:diacylglycerol choline transferases (PDAT). PLC is discussed further below. In *P. tricornutum*, genes coding for a putative PDAT have been predicted (Dolch et al., 2017b), but to our knowledge, not formally characterized. Their roles and possible involvement in the production of TAG substrates deserve investigation.

#### Acyl-coA-Dependent Pathway

DGAT enzymes catalyze the committed step from acyl-CoA and DAG to TAG. Because of its pivotal role in TAG synthesis and of the increasing importance gained by TAGs produced by oleaginous algae like *P. tricornutum* and *Nannochloropsis s.l.* in biotechnology (Ma et al., 2016; Butler et al., 2020), it is no surprise that the number of studies involving DGAT drastically raised in the last decade.

**FIGURE 3:**
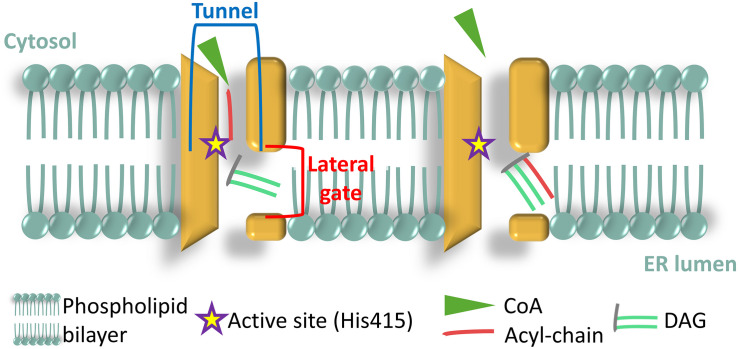
Architecture of the membrane bound *O*-acyl transferase (MBOAT)-core of human diacylglycerol acyltransferase (DGAT1) enzyme (HsDGAT1). Adapted from Sui et al. (2020).

Recently, the human DGAT1 enzyme (HsDGAT1) was structurally characterized (Sui et al., 2020) by cryo-electron microscopy, revealing that the protein contains nine transmembrane helices with the amino-terminus (N-ter) spreading in the cytosol and the carboxyl-terminus (C-ter) in the ER lumen. The HsDGAT1 forms dimers via hydrogen bonds and hydrophobic interaction between the N-termini of each monomer. The dimerization is essential to the acyltransferase activity because N-ter truncated proteins, unable to dimerize, show dramatically reduced activity. The active site, His415 in HsDGAT1 (McFie et al., 2010), is embedded in a tunnel that opens up toward the cytosol formed by four transmembrane (TM) domains (Figure 3), and that serves the acyl-CoA binding function. Laterally, a gate was identified that opens up in between the two membrane sheets (Figure 3) and driving the DAG to the core of the dimer, where the active histidine is located (Sui et al., 2020). Such architectural structure is specific to membrane-bound *O*-acyl transferase (MBOAT) enzymes and was hence named MBOAT-core (Sui et al., 2020). DGAT1 from a green unicellular alga [*Chromochloris zofingiensis*, formerly known as *Chlorella zofingiensis* (Fucikova and Lewis, 2012)] was recently characterized and showed a similar structure of the protein and a crucial role played by its N-ter in protein activity (Xu et al., 2020). DGAT2 family has not yet been characterized in such detail. Murine DGAT2 shows two TM domains and both N- and C- termini in the cytosol (Stone et al., 2006). A highly conserved sequence (HPHG) found in all DGAT2 proteins are the possible catalytic residues (Xu et al., 2018). DGAT3 is the only cytosolic DGAT and was first characterized in peanut (*Arachis hypogaea*), AhDGAT3 (AY875644) (Saha et al., 2006) then in *Arabidopsis thaliana*, AtDGAT3 (At1g48300) (Hernández et al., 2012; Aymé et al., 2018) where it was shown to harbor a [2Fe–2S] cluster, characteristic to some ferredoxins, but whose function is still unknown (Aymé et al., 2018). Moreover, the AtDGAT3 has a chloroplast transit peptide, absent in other higher plant DGAT3.

The protist DGATs have not been yet characterized to such a deep level of detail, and the most recent review focusing solely on algal DGATs dates back to 2012 (Chen and Smith, 2012). A comprehensive review dealing with mainly plant but also algal DGAT and PDAT was recently published (Xu et al., 2018). *P. tricornutum* possesses several DGATs, namely, one PtDAGT1, five PtDGAT2, and a PtDGATX (Cui et al., 2018) or PtDGAT3 (Zhang et al., 2020), with a double wax ester synthase (WS) and DGAT function (Cui et al., 2013, 2018). All the PtDGATs were tested for activity either *in vitro* or by heterologous expression in the *Saccharomyces cerevisiae* quadruple mutant H1246.

*PtDGAT1* (GenBank accession MN061782) was recently reannotated and characterized (Zhang et al., 2020). The authors showed that the original annotation Phatr3_J9794 locus on chromosome 2 lacked part of the N-ter (Guihéneuf et al., 2011). The novel annotation identified a gene composed of a 265-bp 5′-UTR, a 511-bp 3′-UTR, and a 2,271-bp coding sequence interrupted by three introns, encoding for a 756-AA protein (Zhang et al., 2020). PtDGAT1 is a cER (Zhang et al., 2020) membrane-bound protein (eight TM domains were predicted; Guihéneuf et al., 2011) as demonstrated by topology analysis via Western blot (Cui et al., 2018). The novel annotation pointed out the presence in the N-ter of the protein of a pleckstrin homology (PH) domain (Zhang et al., 2020), involved in signaling and membrane binding. The protein shows 55% identity with *Thalassiosira pseudonana* DGT-1 (THAPSDRAFT_261279 on chromosome 2) and 35% with DGAT1 from higher plants. Its function, activity, and specificity were characterized both *in vitro* and by heterologous expression in yeast (Zhang et al., 2020). Among the tested PtDGAT, PtDGAT1 is the most active and presented a slight preference toward mono-unsaturated medium chain FA like C16:1 over saturated medium chain ones (Zhang et al., 2020). A detectable, although reduced, activity was recorded on eicosapentaenoic acid (EPA, C20:5) too. *PtDGAT1* seems to play a crucial role during nitrogen starvation, its expression being highly upregulated in a time-dependent manner at early and late starvation, i.e., when TAG cellular content was highly augmented (Guihéneuf et al., 2011; Cui et al., 2018; Zhang et al., 2020). Overexpression of PtDGAT1 in *P. tricornutum* showed little effect on cell growth, but induced a twofold accumulation of lipids (Zhang et al., 2020). In nitrogen starvation conditions, PtDGAT1 overexpressing (OE) lines redirect carbon from carbohydrates and proteins toward lipid synthesis (Zhang et al., 2020).

The PtDGAT2 family is represented by five protein coding genes in *P. tricornutum*, namely, *PtDGAT2A* (JX469835, Phatr3_49462 on chromosome 22), *PtDGAT2* [JQ837823, Phatr3_49544 on chromosome 22 henceforth referred to as *PtDGAT2B*, as in references (Gong et al., 2013; Haslam et al., 2020; Zhang et al., 2020)], *PtDGAT2C* (JX469836, Phatr3_31662 on chromosome 2), *PtDGAT2D* (JX469837, Phatr3_43469 on chromosome 1), and *PtDGAT2E* (Phatr3_EG00369 on chromosome 19) (Chen and Smith, 2012). *PtDGAT2B* is the only one that rescued the phenotype of the H1246 mutant (Gong et al., 2013; Zhang et al., 2020), had an *in vitro* activity (Zhang et al., 2020), and presented a considerable overexpression in nitrogen starvation (Gong et al., 2013; Zhang et al., 2020).

PtDGAT2B localizes at the cER, like PtDAGT1 (Zhang et al., 2020) and has been more intensively studied (Gong et al., 2013; Haslam et al., 2020; Zhang et al., 2020) compared with other PtDGAT2. It was demonstrated that *PtDGAT2B* expression varies along the growth curve in nitrogen replete condition with a peak at the end of the exponential phase (Gong et al., 2013), while in nitrogen starvation, its expression stays invariably high (Gong et al., 2013; Zhang et al., 2020). Such an expression pattern correlates quite well with the TAG accumulation. Indeed, *PtDGAT2B*-overexpressing lines, accumulate TAGs with no impaired growth in either N-replete nor N-depleted condition (Haslam et al., 2020; Zhang et al., 2020). Coincidently to TAG accumulation, *PtDGAT2B* OE slightly accumulate EPA in N-repletion and show a sharper effect on the accumulation of C16:0 and C16:1 in N-depletion compared with the WT. Analyses of TAG species in the OE lines show an increase in C48 containing TAG, suggesting that PtDGAT2B may prefer C16 species and C16-containing DAG as substrates (Haslam et al., 2020). The very low levels of TAG containing only saturated FA (C48:0) at least partially corroborates previous findings that have shown substrate specificity toward unsaturated FA (Gong et al., 2013; Zhang et al., 2020).

The role played by *PtDGAT2A*, *PtDGAT2C*, and *PtDGAT2D* in the Kennedy pathway remains uncertain because besides not complementing the phenotype of the H1246 mutant neither showing any or very low activity *in vitro*, they are all poorly expressed in both nitrogen replete and deplete conditions (Gong et al., 2013; Zhang et al., 2020).

In *P. tricornutum* genome, the locus Phatr3_J49708, encoding for a predicted protein (XM_002184438, protein ID: XP_002184474), was first isolated from cDNA (Cui et al., 2013) and characterized by heterologous expression in the H1246 mutant and *in vitro* assay (Cui et al., 2013; Zhang et al., 2020). Sequence identity and domain identification revealed that the product of Phatr3_J49708 might be identified as PtDAGT3 by homology to *Acinetobacter calcoaceticus* DGAT3 (AE529086) and because it showed poor similarity to other DGATs found in *P. tricornutum* itself or in other algal species (Cui et al., 2013). PtDGAT3 is localized to the cER (Zhang et al., 2020) unlike the cytosolic AhDGAT3 (Saha et al., 2006), and noteworthy, it presents two domains, namely, a wax ester acyltransferase and acyl-CoA:diacylglycerol acyltransferase domains (Cui et al., 2013). Intriguingly, a few years later, the same predicted protein (XP_002184474) was identified as a novel dual-function PtWS/DGAT and named PtDGATX (Cui et al., 2018). In 2020, the Phatr3_J49708 locus was investigated again and named back as a PtDGAT3 (Zhang et al., 2020). This nomenclatural inhomogeneity should be solved, but it goes beyond the aims of the present review. Nonetheless, henceforth, the Phatr3_J49708 locus product will be referred to as PtDGAT3 (Cui et al., 2013; Zhang et al., 2020). *PtDGAT3* transcript is weakly expressed compared with *PtDGAT1* and *PtDGAT2B* (Cui et al., 2018; Zhang et al., 2020), and along a growth curve, its expression peaks in the middle of the exponential phase. The overexpression of *PtDGAT3* in alga did not impair growth nor photosynthesis efficiency, though it induced a TAG accumulation in both N-replete and N-deplete conditions (Cui et al., 2018; Zhang et al., 2020), which is coherent with the DGAT activity of the protein. Moreover, expression of *PtDGAT3* in the H1246 mutant induced an accumulation, although to a lesser extent, of wax, as expected from the WS domain identified (Cui et al., 2013, 2018).

In conclusion, *P. tricornutum* shows a rather broad toolkit to esterify acyl chains onto a DAG molecule deriving from the Kennedy pathway. Different DGAT proteins presumably exhibit different substrate specificities in order to cope with the wide array of FAs synthesized by *P. tricornutum*. Nevertheless, *in vitro* enzymatic activity tests on C16:0, C16:1, and C20:5 show no striking differences among the PtDGATs (Zhang et al., 2020). It is not excluded, though, that *in vitro* as well as *in vivo* assays tested to date were not adequate to determine PtDGAT’s slight differences in substrate specificity. Moreover, the set of FAs tested *in vitro* was rather reduced. It is not possible to exclude a role of the inactive PtDGAT2s in very specific conditions. DGAT involved in peculiar yet to discover functions to cope with still undetermined conditions may be the reason for a huge gene dose expansion in stramenopiles. Gene duplication in diatom is a relatively frequent event (Parks et al., 2018; Osuna-Cruz et al., 2020) and has been suggested as one of the reasons for the undeniable success of diatoms in the world oceans (Busseni et al., 2019).

The *Nannochloropsis* and *Microchloropsis* genera show the highest number of DGAT genes (Wang D. et al., 2014; Alboresi et al., 2016), namely, two DGAT1 (1A found in *N. oceanica* IMET1, *N. oceanica* CCMP531, *M. gaditana* CCMP526, and CCAP849/5, *N. oculata* CCMP525; 1B found in IMET1, CCMP531, *N. granulata* CCMP529, and CCMP525) and 11 DGAT2 (2A through K) with a very interesting pattern of orthology. *DGAT2* genes are all present in all the strains analyzed, while *DGAT1* genes are not (Wang D. et al., 2014). The strain *N. oceanica* CCMP1779 shows only one *DGAT1* (*NoDGAT1*) and up to 12 *DGAT2* (NoDGTT1 through NoDGTT12) genes (out of which 11 show a EST support) (Vieler et al., 2012b). From phenotype restoration in the TAG-deficient *S. cerevisiae* mutant assay, it emerged that not all the proteins are able to rescue the lipid phenotype, like, e.g., NoGAT1B in *N. oceanica* IMET1 (Li et al., 2016).

Multi-omics analyses performed on several *M. gaditana* (CCAP 849/5, B-31, CCFM-01, and CCMP526) and *N. oceanica* (IMET1, CCAP1779) strains exposed to different stressors showed contrasting results. While some *DGATs* are downregulated in N-starvation, others are upregulated, and others do not change, pointing at the need of more thorough molecular physiology studies on this group of unicellular algae (Corteggiani Carpinelli et al., 2014; Li et al., 2014; Poliner et al., 2015; Alboresi et al., 2016; Xin et al., 2017; Zienkiewicz et al., 2017, 2020; Hulatt et al., 2020; Janssen et al., 2020). Different *DGAT* genes may act on different substrates and/or at different moments along the life cycle of the species, as hypothesized above for *P. tricornutum*. Disentangling the mechanism of each of the DGAT proteins may not only increase our knowledge of stramenopile metabolisms but also can be functional to biotechnological aims.

The non-photosynthetic stramenopiles *Aurantiochytrium limacinum* and *Hondaea fermentalgiana*, although being strong TAG accumulators, possess a reduced DGAT toolkit with two DGAT1 (only one in *H. fermentalgiana*), one DGAT2 and one dual-functioning DGAT/Wes (Dellero et al., 2018a; Seddiki et al., 2018; Morabito et al., 2020), likely involved in the synthesis of wax esters. Although poorly investigated, the possibility of Wes activities may be considered in photosynthetic heterokonts as well.

#### Acyl-CoA-Independent Pathway

Besides *de novo* synthesis via DGATs, TAGs may accumulate through glycerolipid recycling via the activity of a so-called phospholipid:diacylglycerol acyltransferase, or PDAT (Dahlqvist et al., 2000; Ma et al., 2016; Zulu et al., 2018; Falarz et al., 2020). The acyl-CoA-independent pathway consists in the transfer of an FA from the *sn*-2 position of a membrane lipid (usually PC, in non-photosynthetic organisms) to the *sn*-3 position of the glycerol backbone of DAG. This pathway was far less investigated than the acyl-CoA-dependent route in algae. Whereas in non-photosynthetic models, such as yeast, PDAT is located at the ER and using phospholipid as an acyl-donor, functional analysis of *Chlamydomonas reinhardtii* PDAT suggested a role in transferring FA groups from plastid membrane lipids as well (Yoon et al., 2012). By contrast with the vast number of DGATs, only one copy of PDAT is usually encountered in photosynthetic stramenopiles, such as *Nannochloropsis s.l.* (Dolch et al., 2017b; Nobusawa et al., 2017). On the basis of transcriptomic results, the PDAT pathway was suggested to be responsible for TAG accumulation in *M. gaditana* CCAP 849/5 alongside the DGAT-dependent synthesis (Mus et al., 2013; Yang et al., 2013; Alboresi et al., 2016). It seems that some physiological conditions may be in favor of a prominence of the DGAT- or the PDAT-dependent routes; however these conditions have not yet been characterized, and the level of possible redundancy/compensation is unknown. Thus, on the one hand, ^13^C isotopic labeling results showed that at least in *M. gaditana* strain CCFM-01, most of the FAs in TAGs stemmed from *de novo* synthesis (Janssen et al., 2019a). On the other hand, in a knockdown (KD) line of an elongase converting 16:0-CoA into 18:0-CoA in the cytosol of *M. gaditana*, disturbing membrane glycerolipid composition, only the *MgPDAT* gene proved to be upregulated and likely responsible for the observed accumulation of TAG (Dolch et al., 2017b). The control of the acyl-CoA-independent pathway needs to be addressed in stramenopiles, as well as possible redundancy with the acyl-CoA dependent routes. Since PDAT allows a connection between TAG production and membrane lipid turnover, a role of the reorganization of cellular membranes needs also to be investigated.

### Stepwise Formation of Lipid Droplet Subpopulations in *Phaeodactylum*

#### Lipid Droplet Biogenesis Steps

Stepwise LD formation has been mostly described in non-photosynthetic organisms (Walther et al., 2017; Nettebrock and Bohnert, 2020; Renne et al., 2020) even though recent review articles have underlined some specificities of plastid-containing organisms (Chapman et al., 2019; Ischebeck et al., 2020; Leyland et al., 2020a). Cytosolic LD biogenesis is usually described as an ER process. Briefly, TAG and sterol ester synthesis occurs between the two leaflets of the ER membrane leading to the formation of lenses once they reach a certain concentration (Choudhary et al., 2015; Thiam and Forêt, 2016). After lense growth, complex mechanisms lead to the budding of an LD from the ER membrane at specialized domains. Data from yeast as well as animals suggest that such domains are sites of both LD and peroxisome biogenesis (Joshi et al., 2018; Wang et al., 2018), thus linking the two processes. LD budding is mostly described in biophysical terms. Specific lipid composition at LD budding sites locally changes the membrane curvature (Ben M’barek et al., 2017; Choudhary et al., 2018; Santinho et al., 2020), and the introduction of an imbalance in surface tensions between the two membrane leaflets is essential for directional budding (Chorlay and Thiam, 2018; Chorlay et al., 2019). The role of proteins in the budding is therefore important to regulate local lipid composition and locally modify surface tension, as well as to stabilize the junction between the nascent LD and the ER and to ensure the unidirectional filling of the LD. Only a few proteins involved in LD biogenesis have been identified in all eukaryotic models.

First identified in humans as responsible for the Bardinelli–Seip congenital lipodystrophy (Magré et al., 2001), the seipin protein is a key player in LD biosynthesis in all organisms in which it has been investigated (Chapman et al., 2019; Nettebrock and Bohnert, 2020; Renne et al., 2020). Seipin proteins localize in discrete ER domains that define LD biogenesis sites (Wang C.-W. et al., 2014; Wang et al., 2016; Grippa et al., 2015; Salo et al., 2016). Seipin proteins contain at least two transmembrane domains that anchor them in the ER membrane. The central luminal part is involved in seipin’s oligomerization, thus forming a channel-like structure that stabilizes the ER–LD junction (Binns et al., 2010; Sui et al., 2018; Yan et al., 2018) and is essential to ensure unidirectionality of the TAG and/or sterol–ester flow from the ER to the LD (Salo et al., 2019). Moreover, in humans, such channel-like domain can interact *in vitro* with anionic phospholipids, in particular, PA (Yan et al., 2018). If confirmed *in vivo*, such an interaction could contribute to local changes in the ER membrane composition that is involved in budding, as depicted above. Finally, seipin plays a role in metabolism regulation, through its interactions with lipid biosynthesis enzymes (Boutet et al., 2009; Sim et al., 2013; Talukder et al., 2015; Pagac et al., 2016; Su et al., 2019). While yeast and animals possess only one seipin isoform, three have been identified in *Arabidopsis thaliana* (Cai et al., 2015), and it has been shown that the N-terminal tail of seipins is involved in LD size control. Seipin isoforms are supposed to have distinct functions depending on the tissue, and all of the three are required for proper LD biogenesis in embryos (Taurino et al., 2018). In addition to lipid metabolism enzymes as mentioned above, seipin interacts with different proteins depending on the organism. In yeast, the seipin homolog Fld1 function depends on its interaction with Ldb16 (Wang C.-W. et al., 2014). Yet this seems to be yeast specific as no homolog of Ldb16 has been identified in other eukaryotes. The expression of human seipin is sufficient to rescue the double *Fld1/Ldb16* KO (Wang C.-W. et al., 2014). The Fld1/Ldb16 complex interacts with Ldo16 and Ldo45 (Lipid Droplet Organization proteins) (Eisenberg-Bord et al., 2018). This interaction is conserved in mammals for which Promethin/TMEM159/LDAF1 (lipid droplet assembly factor) has been identified as the Ldo45 homolog (Castro et al., 2019; Chung et al., 2019). In both yeast and humans, the abovementioned interaction of seipin may determine the sites of LD formation (Bohnert, 2020). Recently, the yeast phosphatase Nem1 has also been identified as an interactor of seipin, playing a major role in the recruitment of TAG synthesis enzymes to LD biogenesis sites (Choudhary et al., 2020). In *Arabidopsis thaliana*, seipins 2 and 3 interact with the vesicle-associated membrane protein (VAMP) -associated protein VAP27, and this interaction proved crucial for LD formation, although the underlying mechanism still needs to be investigated (Greer et al., 2020). A seipin homolog has been identified in *P. tricornutum* (Lu et al., 2017) and confirmed as an important factor in LD biogenesis as its overexpression leads to TAG accumulation and increased LD formation. By sequence homology, the *Thalassiosira pseudonana* locus *THAPSDRAFT_1237* could encode a seipin homolog. Conversely, no homologs have been found in *Nannochloropsis s.l.*

Other factors have been involved in LD biogenesis in Opisthokonts and Viridiplantae. Interestingly, some seem to be specific to one or the other phylum, e.g., the FIT proteins that play a major role in Opisthokonts (Renne et al., 2020) could not be identified in Viridiplantae nor in the *P. tricornutum* genome. Likewise, the LD major structural proteins identified in stramenopiles (see below) are different from perilipins found in Opisthokonts or oleosins found in Viridiplantae.

**FIGURE 4:**
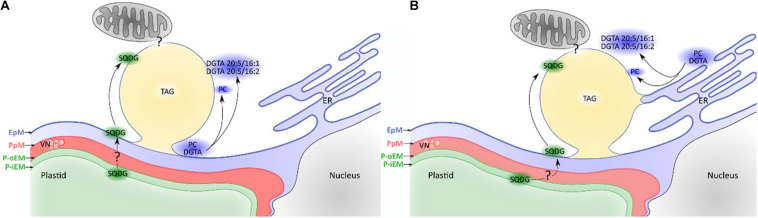
Different hypotheses regarding lipid droplet (LD) biogenesis in *Phaeodactylum tricornutum*. EpM, epiplastidial membrane; PpM, Periplastidial membrane; P-oEM, plastid outer envelope membrane; P-iEM, plastid inner envelope membrane; ER, endoplasmic reticulum; VN, vesicular network; SQDG, sulfoquinovosyldiacylglycerol; PC, phosphatidylcholine; DGTA, diacylglycerylhydroxymethyl-*N*,*N*,*N*-trimethyl-β-alanine; TAG, triacylglycerol). **(A)** Lipid droplets emerge from the EpM. The EpM membrane contains PC and specific DGTA species, but also SQDG. The abundance of C16:2 on the betaine lipid DGTA found in the lipid droplet monolayer (Lupette et al., 2019) supports a plastid emerging hypothesis as this fatty acid is major in the plastid but very minor in endomembranes (Abida et al., 2015). The origin of SQDG in the EpM is unknown and may be explained either by an export from the plastid or a synthetic pathway located in the EpM. **(B)** Lipid droplets form at an interface between the ER and the EpM. PC and DGTA can originate from both membranes, while SQDG comes from the EpM as described above. The peculiar DGTA composition (Lupette et al., 2019) suggests that only certain species are present at LD biogenesis sites although the sorting mechanism is unknown.

Another major peculiarity of stramenopiles is the biogenesis site of LD. As mentioned above, the ER is widely considered as the site of LD biogenesis. Yet, studies in the green alga *C. reinhardtii* suggest that the chloroplast and the ER are jointly involved in LD biogenesis (Fan et al., 2011; Goodson et al., 2011; Tsai et al., 2015). The architecture of the secondary plastids is unique, and the outermost membrane, the EpM, is in continuity with the ER (see above). Electron microscopy analyses have shown very close contacts between the plastid and the LD in *P. tricornutum* (Flori et al., 2016; Lupette et al., 2019; Jaussaud et al., 2020). Moreover, the lipid monolayer surrounding the LD includes the plastid glycolipid SQDG as well as PC and the betaine lipid DGTA (Lupette et al., 2019) (see above) that is considered to be synthesized in the ER. Two major DGTA species are found in the LD, namely, DGTA C20:5/C16:1 and C20:5/C16:2 (Lupette et al., 2019), the latter being a minor DGTA species in *P. tricornutum* (Abida et al., 2015; Lupette et al., 2019). While C20:5 biosynthesis occurs in the ER (Dolch and Maréchal, 2015), C16:2 is a major FA in MGDG and DGDG but is mostly absent from ER lipids (Abida et al., 2015). This particular composition thus suggests an interplay between ER and plastid functions, and two main hypotheses could explain it (Figure 4). First, the EpM could be the site of LD biogenesis instead of the ER (Figure 4A). EpM is continuous with the ER, and hence, it may exert some classical ER functions (Flori et al., 2016). Alternatively, as hypothesized in *C. reinhardtii*, LD biogenesis could occur through links with both the plastid and the ER (Figure 4B). In support to the latter hypothesis, electron microscopy inspections show association of the LD with endomembranes in *P. tricornutum* (Lupette et al., 2019). Another corroboration to this hypothesis is supplied by the tight links between the LD and the ER in *Fistulifera* sp. (Nojima et al., 2013), suggested by proteomics analysis of oil bodies.

Contact points and molecular transfers between LD and mitochondria may also occur and need to be characterized. They may, in particular, be important for TAG degradation (Jallet et al., 2020).

#### Several Populations of Lipid Droplets Observed Within *Phaeodactylum tricornutum* Cells Under Nitrogen Deprivation

While proteomics and lipidomics analyses are performed on the global population of lipid droplets, there may be heterogeneity among them. Indeed, the accumulation of LD upon nitrogen starvation in *P. tricornutum* involves several populations of LD biogenesis (Jaussaud et al., 2020). The initial population P1 LD grows in size until a maximal size is reached at day 3, then two additional populations formed. While P3 LD also expand, although not as much as P1 LD, P2 LD stays small. The maximal size of P1 and P3 LD is increased in mutants with a larger cell size, suggesting that the rigid cell wall of *P. tricornutum* is a determinant limiting factor for LD expansion in WT cells (Jaussaud et al., 2020). This may be a difference among stramenopiles. While *P. tricornutum* does not increase in cell size, either phosphate or nitrogen deprivation induces a cell volume increase in *N. oceanica* (Mühlroth et al., 2017) or *Nannochloropsis* sp. PJ12 (Liang et al., 2019b). Thus, cell volume increase may be a common trait under nutrient stress in *Nannochloropsis s.l.* Interestingly, the size of P2 LD in *P. tricornutum* is not changed in larger cells (Jaussaud et al., 2020), raising the possibility that the composition of P2 LD population is different from that of P1 and P3. Additionally, P1 LD growth is concomitant with a general increase in the cell lipid content, suggesting that TAG filling P1 LD population, mostly come from *de novo* synthesis. On the other hand, the emergence of P2 and P3 population correlates with a decrease in polar glycerolipids, ventilating the hypothesis that most of their TAG content may derive from membrane lipid recycling. It must be noticed that the fine sequence of biogenesis of LD subpopulation deciphered in the Pt1 ecotype of *P. tricornutum* (Jaussaud et al., 2020) was not detected in the Pt4 ecotype (Leyland et al., 2020b). Further studies are needed to disentangle the origin, composition, and functions of the different populations of LD.

## Lipid Droplet Formation in Response to Nitrogen and Phosphate Starvation

Accumulation of LDs in response to nutrient limitation is often correlated with a major lipid remodeling, a decrease in photosynthetic activity, and a much slower growth (Meng et al., 2017; Janssen et al., 2019b). Indeed, shortage of nitrogen and phosphate has striking consequences on cell phenotypes. Nitrogen is an essential component of nucleobases, proteins, and many glycerolipids (e.g., PC, DGTS, and PE), while phosphate is required in nucleic acids, sugar molecules, phosphoproteins, and phospholipids. Phosphate may also be a critical element for such molecules as adenosine phosphates or NADPH, the “energy” currencies of the cell to perform its metabolism. The metabolism of all organisms is therefore finely adjusted and tuned in response to the availability of these nutrients.

### An Intense Lipid Remodeling Is Triggered Upon Nitrogen or Phosphate Starvation

**FIGURE 5:**
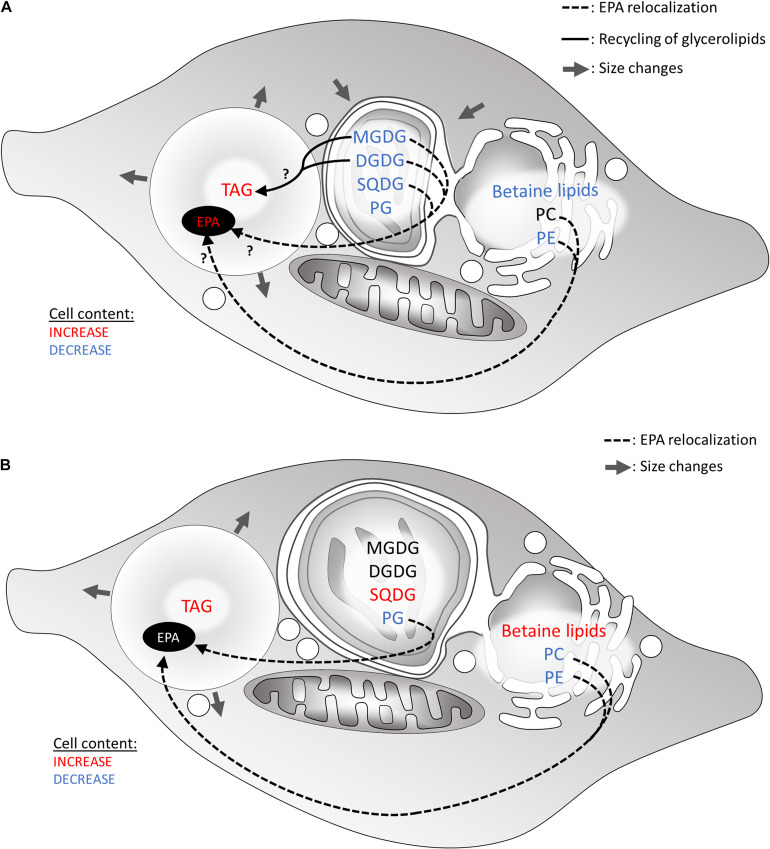
Hypothetical recycling of products of membrane glycerolipid breakdown for the formation of TAG in heterokonts subjected to a nitrogen or phosphate shortage. **(A)** Lipid remodeling under nitrogen starvation. Most glycerolipids are degraded, affecting the plastid size. **(B)** Lipid remodeling under phosphate starvation. In both scenarios, FA released from membrane lipids may be consumed via the β-oxidation pathway in the mitochondrion. Alternatively, down-products can be recycled to form TAG. Arrows show hypothetical conversions of membrane lipid down-products in the production of TAG, as well as in the increase in very long-chained PUFA (e.g., 20:5) in LDs. Glycerolipid conversions occur to rescue phospholipid degradation. DGDG, digalactosyldiacylglycerol; EPA, eicosapentaenoic acid; MGDG, monogalactosyldiacylglycerol; PC, phosphatidylcholine; PG, phosphatidylglycerol; SQDG, sulfoquinovosyldiacylglycerol; TAG, triacylglycerol.

Stress-induced lipid remodeling corresponds to changes in the proportions of glycerolipid classes, in the FA contained in each class, and sometimes includes modification of subcellular location (Jaussaud et al., 2020). Upon nitrogen deprivation, the level of all glycerolipid classes decreases in *Nannochloropsis s.l.* (Simionato et al., 2013; Meng et al., 2017). Only in *Nannochloropsis* sp. PJ12 were PC and PE found to possibly increase during nitrogen deprivation (Liang et al., 2019b). The authors suggest that the increase in PC and PE could be due to their important role in acyl editing, providing a pool of elongated and desaturated FAs (Schneider and Roessler, 1994; Benning, 2009). In *P. tricornutum*, MGDG, PG, and PE decrease, while other lipid classes are stable (Abida et al., 2015; Figure 5).

Phosphate deprivation is apparently a less severe stress compared with nitrogen deprivation, possibly due to the presence of intracellular storage forms; nonetheless, it also leads to an intense lipid remodeling. Phosphate deprivation triggers a breakdown of membrane phospholipids (Abida et al., 2015; Cañavate et al., 2017a,b; Mühlroth et al., 2017; Liang et al., 2019a). Such a decline designates phospholipids as a possible substantial P storage form. Whereas in plants, a PC-to-DGDG appeared as a general phosphorus-saving process, no such mechanism could be evidenced in any stramenopile model studied to date (Abida et al., 2015). Rather, the proportion of another class of non-phosphorus lipid, i.e., betaine lipids, increases (Abida et al., 2015; Cañavate et al., 2017a,b; Mühlroth et al., 2017; Liang et al., 2019a; Meng et al., 2019); it was suggested that phospholipids were replaced by betaine lipids in membranes (Abida et al., 2015; Cañavate et al., 2017a; Mühlroth et al., 2017; Meng et al., 2019), as also suggested in other phytoplankton species (Van Mooy et al., 2009; Cañavate et al., 2017b). This specific conversion of PC-to-betaine lipids has been proposed in several studies (Abida et al., 2015; Iwai et al., 2015; Meng et al., 2019). In particular, in *N. oceanica*, DGTS replaces PC and becomes central during lipid remodeling as a platform for C18 desaturation, maintaining eicosapentaenoic acid (EPA or C20:5) biosynthesis and providing EPA for plastid glycolipids synthesis (Meng et al., 2019), as it was already suggested during nitrogen deprivation (Meng et al., 2017). The PC-to-betaine lipid switch is considered as specific to the endomembrane system.

By the same token, in plastid membranes, phosphate deprivation triggers a striking increase in SQDG proportion in *P. tricornutum* and *N. oceanica* (Abida et al., 2015; Cañavate et al., 2017b; Mühlroth et al., 2017), while it decreases in *Nannochloropsis* sp. PJ12 (Liang et al., 2019a), and is stable in *M*. *gaditana* (Cañavate et al., 2017b). In *P. tricornutum* and *N. oceanica*, the decrease in PG is concomitant with the increase in SQDG (Abida et al., 2015; Cañavate et al., 2017b; Mühlroth et al., 2017), reflecting a specific PG-to-SQDG conversion (Abida et al., 2015; Iwai et al., 2015; Cañavate et al., 2017b). This conversion is reminiscent to that established in other organisms, such as *Arabidopsis* under phosphate deprivation (Nakamura, 2013) and has been reported in other phytoplankton exposed to a phosphate shortage as well (Van Mooy et al., 2009; Cañavate et al., 2017b).

Degraded phospholipids can provide building blocks for TAG accumulation. FA released from membrane glycerolipids may be consumed via the β-oxidation pathway in the mitochondrion. Alternatively, down-products can be recycled to form TAG. In particular, PC is supposed to be involved in acyl-CoA-independent synthesis of TAG as inferred by the activation of the Land’s cycle and the PDAT enzymes upon phosphate depletion (Mühlroth et al., 2017). Hypothetical flows of materials released from membrane lipid breakdown to TAG are summarized in Figure 5.

### Eicosapentaenoic Content in Lipid Class, a Signature to Trace Lipid Remodeling

Under nitrogen as well as phosphate deprivations, the EPA level in total lipid decreases in *Nannochloropsis s.l.* (Simionato et al., 2013; Abida et al., 2015; Meng et al., 2015; Liang et al., 2019a,b), while it is stable in *P. tricornutum* (Abida et al., 2015). However, EPA accumulation in TAG increases in both taxa (Abida et al., 2015; Meng et al., 2017; Janssen et al., 2019b; Liang et al., 2019b). Due to the interest toward EPA for several industrial applications, the source of EPA during lipid remodeling has been further investigated in order to find new ways to improve EPA content in TAG (Simionato et al., 2013; Abida et al., 2015; Jia et al., 2015; Meng et al., 2015; Liang et al., 2019b). For example, contribution of galactolipids to TAG synthesis is emphasized by the probability that, under nitrogen deprivation, the EPA present in TAG partly stems from galactolipid recycling, based on FA balance between these lipid classes (Simionato et al., 2013; Abida et al., 2015; Jia et al., 2015; Meng et al., 2015; Liang et al., 2019b; Figure 5). In *P. tricornutum* under phosphate deprivation, phospholipids are supposed to also provide part of the EPA in TAG molecular species (Abida et al., 2015), and this was also hypothesized in *M. gaditana* under nitrogen deprivation (Janssen et al., 2019a). In *N. oceanica* under normal growth conditions, PE and DGTS seem to provide EPA to other lipids, in particular to MGDG (Vieler et al., 2012b; Meng et al., 2017). However, it was not shown whether translocation of EPA to TAG under nitrogen deprivation originated from PE and DGTS and/or from MGDG (Meng et al., 2017). In *P. tricornutum*, the role of EPA source is apparently played by PC (Mühlroth et al., 2013). By monitoring EPA within membrane glycerolipids and TAG, various routes for very long-chain polyunsaturated FAs are highlighted within glycerolipid metabolism, and an important future challenge is to decipher the molecular determinants of each route, and their controlling and regulatory mechanisms.

### Enzymes Involved in Lipid Reshuffling Are Upregulated at the Transcriptional Level

Transcriptomic analyses revealed that lipases involved in lipid remodeling were activated during nitrogen and phosphate deprivation. In *N. oceanica* under nitrogen shortage, a homolog of the *Arabidopsis* galactolipase *PSD1* is upregulated, coincidently with degradation of MGDG (Li et al., 2014). Diverse phospholipases A, C, and D (PLA, PLC, and PLD, respectively) were also identified in both *P. tricornutum* and *Nannochloropsis s.l.* In *P. tricornutum*, under both nitrogen and phosphate starvation, several *PLC* and *PLD* isoforms were upregulated (Alipanah et al., 2015, 2018; Cruz de Carvalho et al., 2016; Remmers et al., 2018). The upregulated *PLC* isoforms are all predicted to encode for PI-specific PLCs in the case of the phosphate deprivation (Cruz de Carvalho et al., 2016; Alipanah et al., 2018). In *N. oceanica* under nitrogen deprivation, several genes encoding for putative patatin and one putative lysophospholipase were upregulated under nitrogen deprivation (Li et al., 2014; Jia et al., 2015). Meng et al. (2019) did not find any upregulated genes related to phospholipid degradation under phosphate deprivation in *N. oceanica* (Meng et al., 2019). Conversely, Mühlroth et al. (2017) found three patatin-like PLAs together with two glycerophosphoryldiester phosphodiesterases (GDPDs) upregulated, which could lead to the release of FAs and G3P from phospholipids, leading to the complete recycling of phospholipids in *N. oceanica* (Mühlroth et al., 2017).

Key enzymes of the Land’s cycle were also found to be upregulated. Both phosphate and nitrogen deprivation induced upregulation of one putative PDAT in *Nannochloropsis s.l.* and *P. tricornutum* (Li et al., 2014; Alipanah et al., 2015, 2018; Jia et al., 2015; Cruz de Carvalho et al., 2016; Mühlroth et al., 2017). One LPCAT and one PLA2 were also overexpressed under phosphate deprivation in *N. oceanica* (Mühlroth et al., 2017), completing the Land’s cycle.

## More Lipid Droplet Function in the Cell: Hypothesis Deduced From Proteomic Studies

As mentioned above, TAG production and storage are induced under stress conditions, which results in cell cycle slowdown or arrest. The energy excess produced by photosynthesis that still works during the first phases of stress is stocked and constitutes a pool of carbon and energy available upon stress release. However, in stramenopiles, as in other organisms (Welte, 2015), LD function is not limited to an energy reservoir as can be hypothesized from proteomics analyses.

### The Protein Equipment of Lipid Droplets, Insights Into Lipid Droplet Function and Connection to Other Organelles

#### Identification of Major Structural Lipid Droplet Proteins

Several proteomics analyses have been performed in different stramenopiles, e.g., *Nannochloropsis oceanica* (Vieler et al., 2012a), *Fistulifera* sp. (Nojima et al., 2013; Nonoyama et al., 2019), and *P. tricornutum* (Yoneda et al., 2016; Wang et al., 2017; Lupette et al., 2019; Leyland et al., 2020b). Purification of LDs consisted in (i) treatment of cells to nutrient starvation (mostly nitrogen deprivation) to trigger LD formation, (ii) cell disruption, and (iii) LD isolation, generally through sucrose density centrifugation. This classical method was particularly improved in proteomic analysis in *P. tricornutum* (Lupette et al., 2019) where it greatly reduced contamination from plastid and other cellular compartments.

**FIGURE 6:**
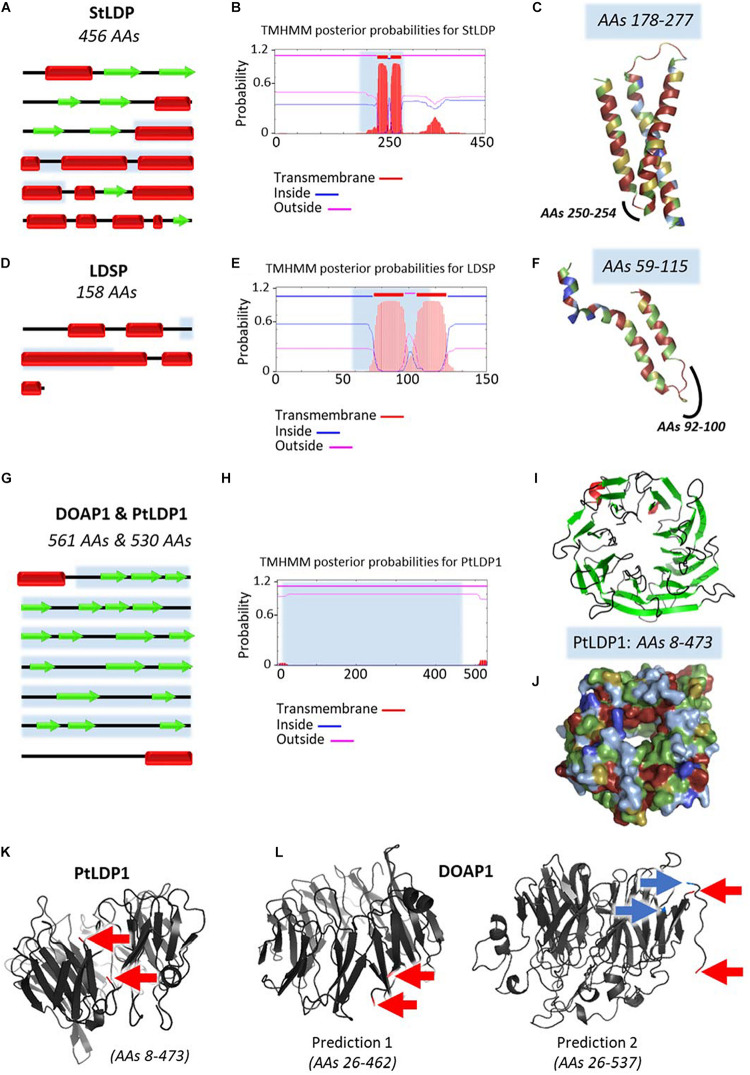
Predicted structures of major lipid droplet proteins found in stramenopile models. Analysis of predicted structures and hydrophobic regions in **(A–C)** StLDP from *Phaeodactylum tricornutum*, **(D–F)** LDSP from *Nannochloropsis s.l.*, and the highly similar **(G–L)** DOAP1 and PtLDP1 from *Fistulifera solaris* and *P. tricornutum*, respectively. **(A,D,G)** Secondary structure representations adapted from results obtained with Jpred4 prediction server (http://www.compbio.dundee.ac.uk/jpred/). Beta sheets are represented with red rectangles, and alpha helices with green arrows. **(B,E,H)** Predictions of transmembrane helices obtained with TMHMM prediction server (http://www.cbs.dtu.dk/services/TMHMM/). **(C,F,I,J,K,L)** Predicted tertiary structures obtained with Phyre^2^ (Protein Homology/analogy Recognition Engine V2.0) prediction server (http://www.sbg.bio.ic.ac.uk/∼phyre2/html/page.cgi?id = index) in ribbon diagrams **(C,F,I,K,L)**, and surface representation **(J)**. Obtained Phyre^2^ predictions span only a portion of the proteins, first and last amino acids (AAs) are specified in the figures. These regions are indicated in blue in the secondary structures representations **(A,D,G)** and TMHMM results **(B,E,H)**. Hydrophobicity level of AAs is represented with a color code in **(C,F,I,J)**, ranging from dark blue for very hydrophobic AAs, light blue for hydrophobic AAs, green for poorly hydrophobic or hydrophilic AAs, yellow for hydrophilic AAs, and red for very hydrophilic AAs. These predictions show that StLDP and LDSP have a hairpin structure, with the hairpin being enriched in hydrophobic residues. For DOAP1 and PtLDP1, a beta barrel structure bordered with two alpha helices is predicted. The membrane-spanning sequences of the beta barrel do not seem as enriched in hydrophobic residues as the alpha-helices of the hairpin structures of StLDP and LDSP, as already observed in the literature for these structures (Tamm et al., 2004; Wimley, 2009). The alpha helices potentially anchor the protein in the membrane as suggested by the TMHMM results. These alpha helices seen in the secondary structure are not present in the predicted tertiary structure due to incomplete models. To infer the position of the alpha helices regarding the beta barrel, first and last amino acids of the spanned regions are indicated with red arrows for one PtLDP1 model **(K)** and two possible DOAP1 models **(L)**. DOAP1 model spanning the AAs 26–537 offers a more complete model than the model covering the AAs 26–462. However, a break in the predicted tertiary structure appears in this model between the two AAs indicated with blue arrows. The two alpha helices appear to be close to each other. However, it is not possible to conclude if the helices are in the same direction. AAs, amino acids; DOAP1, diatom oleosome-associated protein1; LDSP, lipid droplet surface protein; PtLDP1, *P. tricornutum* lipid droplet protein1; StLDP, stramenopile lipid droplet protein.

With the noticeable exception of *Fistulifera* sp., proteomics analysis revealed the presence of quantitatively major LD proteins. In *Nannochloropsis* sp., the main protein was named the lipid droplet surface protein (LDSP, accession number AFB75402) and immunodetected in four *Nannochloropsis s.l.* species among which are *M. gaditana* and *N. oceanica* (Vieler et al., 2012a). The LDSP had no previously assigned function and was first hypothesized to serve as a major structural component (Vieler et al., 2012a). It was later on shown to be likely involved in LD degradation through binding to autophagy-related protein 8 (ATG8) (Zienkiewicz et al., 2020). A detailed comparison of stramenopile LDPs characterized to date is provided in Figure 6.

For *P. tricornutum*, the two first LD proteomic analyses unexpectedly identified distinct sets of proteins. The first analysis identified nine proteins, among which the most abundant is the Stramenopile-type lipid droplet protein (StLDP, accession number XP_002183367), named based on the homology found in six other stramenopiles including *M. gaditana* (Yoneda et al., 2016). StLDP possibly plays a role in the maintenance, distribution, and degradation of TAG in LDs (Yoneda et al., 2016). The second analysis identified five proteins, with PtLDP1 (accession number XP_002178287) being the most represented (Wang et al., 2017). OE and KD of PtLDP1 led to changes in lipid content, LD size, and relative expression levels of key genes involved in TAG and FA biosynthesis (Wang et al., 2017), suggesting an important role in LD biogenesis and regulation of lipid synthesis. Interestingly, PtLDP1 shares high similarities with one of the LD protein identified in *Fistulifera* sp., DOAP1 (accession number BAO47264) (Nojima et al., 2013; Maeda et al., 2014; Wang et al., 2017). DOAP1 and PtLDP1 both possess a terminal signal peptide, and further analyses suggested that DOAP1 may localize to the ER prior to the transfer to LDs (Maeda et al., 2014). The difference between the two studies may be due to different nitrogen starvation conditions (6 vs. 2 days), corresponding to late and early LD formation, respectively (Yoneda et al., 2016; Wang et al., 2017). Protein equipment of nascent and budding LDs is indeed thought to differ from that of mature LDs. In the study performed by Lupette et al. (2019) on mature LDs (7 days of nitrogen starvation), StLDP and PtLDP1 were both recorded in the LD proteome, although no major LD protein was pointed out. The presence of StLDP was further confirmed in a more recent analysis of LD proteome in the Pt4 ecotype of *P. tricornutum* (Leyland et al., 2020b).

LD-associated proteins have been classified according to their structure and provenance (Kory et al., 2016). Class I proteins are inserted in the membrane through a hydrophobic hairpin structure and access the LD surface *via* the ER, while class II proteins access the LD from the cytosol, using other hydrophobic domains. Both LDSP and StLDP are class I proteins, while PtLDP1 and DOAP1 are class II proteins (Figure 6).

LDSP, StLDP, and PtLDP1 all contain hydrophobic sequences harboring a proline-rich region (Figure 6), similar to the proline knot motif found in plant oleosins (Abell et al., 2004). *LDSP*, *StLDP*, and *PtLDP1* gene expression can be induced by stress conditions leading to lipid accumulation (Yoneda et al., 2016; Wang et al., 2017; Zienkiewicz et al., 2020), and their function is related to the modulation of LD size (Vieler et al., 2012a; Yoneda et al., 2016; Wang et al., 2017).

Altogether, it seems that like in other eukaryotic lineages, it is difficult to identify major LDPs conserved at the whole clade level. This diversity of LPDs is puzzling. Either LDPs are simply protective proteins, which assemble into a subspherical “shield” preventing free interaction between the LD core and the cytosol, possibly acting as a docking surface for more peripheral proteins, or LDPs may act in dynamic processes as well, for metabolic or signaling processes that need to be uncovered.

#### Mining *Phaeodactylum tricornutum* Lipid Droplet Protein Equipment

As mentioned above, very low contamination was observed in the mature LD proteome produced by Lupette et al. (2019), leading to highly pure LDs and the identification of a core proteome made of 86 proteins. LD function depends on its protein equipment. Based on gene ontology, LD-associated proteins can be divided in seven large functional and structural groups: metabolism, membrane organelle, chaperones/protein folding/post translational modification/quality control, cytoskeleton, genomic information processing, plastoglobules, and “unknown” (Lupette et al., 2019). Plastoglobule-specific proteins probably prove the unavoidable contamination of the purified LD faction by plastid lipid bodies.

Proteins involved in metabolism were mainly cytosolic enzymes participating in the glycolytic and pentose phosphate pathways (Lupette et al., 2019). Others were mitochondrial components of the Kreb’s cycle, and two were the plastidial carbonic anhydrase and acetyl-coA carboxylase (ACC1) (Yoneda et al., 2016; Lupette et al., 2019). The latter two enzymes reveal the proximity of the LD to the plastid, i.e., the compartment devoted to the initiation of FA biosynthesis.

Proteins related to membrane organelles stemmed mainly from the endomembrane, and support a connection between the LD and cytosolic vesicles. The presence of two subunits of the coat protein complex 1 (COP1) coatomer (COPA and COPBETA2) were identified (Lupette et al., 2019). COP1 complex participates in the retrograde transport of vesicles from the *trans*-Golgi network to the ER and in intra-Golgi transport, and depends on ARF1 to be operational (Serafini et al., 1991), which was identified in the unfiltered LD proteome (326 proteins) (Lupette et al., 2019). Two components of the clathrin vesicle coating system (CHC and PTAP1/2BETA) were also identified (Lupette et al., 2019). Clathrins participate in the transfer of proteins outside *trans* Golgi network (Jürgens, 2004). These components could be linked with the presence in the unfiltered proteome of SNARE proteins, whose function is necessary for fusion of transport vesicle with target membrane (Tanaka et al., 2015), and which play a role in COP1 transport (Lupette et al., 2019). Finally, a proton pump found in endomembrane vesicle, V-ATPase, and an ABC-transporter, are also present (Lupette et al., 2019). SLC25A4 (an adenine nucleotide translocator) is located in the inner mitochondrial envelope membrane, supporting a possible connection of the LD with the mitochondrion (Lupette et al., 2019). Some of these protein components were confirmed in a more recent proteomic study of LD purified from the Pt4 ecotype of *P. tricornutum* (Leyland et al., 2020b).

No proteins were identified as originating from the plastid, or more exactly the EpM in the LD proteome of Lupette et al. (2019). Nonetheless, predictions suggest that five out of the 10 proteins classified as “unknown” possess either a signal peptide or a chloroplast transit peptide (cTP) (Lupette et al., 2019). Connection between the LD and the endomembrane, the mitochondria, and the EpM were further supported with electron microscopy images (Flori et al., 2016).

Several proteins were related to mRNA translation and control of misfolded proteins. On one hand, chaperones including heat shock proteins (HSP, HSP70A, HSP90, and HSP40), ribosomal subunits (40S and 60S), and components of the RNA translation machinery (EF1A, EF2, and EIF4A) were identified (Yoneda et al., 2016; Lupette et al., 2019). On the other hand, proteins involved in post translational modification such as ubiquitination (UBI3 and PUB39) and phosphorylation (STK and EPK2) were found, as well as proteins involved in the endoplasmic reticulum-associated degradation (ERAD) system (CDC48 and BIP) (Lupette et al., 2019). Consistent with the presence of ubiquitination proteins, four 26S proteasome proteins are also present in the LD proteome (Lupette et al., 2019). Altogether, the presence of the aforementioned proteins supports the hypothesis for the LD as a potential site for mRNA storage and as platform for specific synthesis and folding of proteins at the periphery of the LD.

Histones H2B-1B, H3-1C, H3.3, and H4-1B were identified in the LD proteome as well. Histone proteins are involved in genome information processing, and their presence in the LD suggests that the LD plays a role in storing and protecting specific proteins during starvation, i.e., a context of intense protein degradation (Lupette et al., 2019). Protection of nucleosome components could prepare for a quick recovery of cell division and chromatin packaging upon the stressing condition (e.g., nutrient starvation) release.

### Getting Ready to Recover From Stress

When favorable growth conditions are restored, the energy stored in LDs is used to reactivate cell cycle and photosynthesis activity. In algae, several forms of lipophagy (lipid degradation by autophagy) may be involved in LD mobilization. Macrolipophagy is characterized by the formation of an autophagosome triggered by autophagy-related (ATG) proteins (Ward et al., 2016). The autophagosome carries, within a double membrane, molecules or damaged organelles to the vacuole for degradation. Fusion of the autophagosome with the vacuole also requires ATG proteins. By microlipophagy, the components to degrade are delivered to the vacuole by invagination of the tonoplast (Ward et al., 2016). Finally, chaperone-mediated autophagy (CMA) is a process by which proteins directly enter the vacuole *via* a translocation complex localized on the vacuole membrane by the heat shock cognate protein 70 (HSC70) and other cytosolic chaperones (Yim and Mizushima, 2020). Autophagy components and chaperones were regularly detected in stramenopile LD proteomes.

In *N. oceanica*, the ATG8 expression level is induced directly after the transition from nitrogen-depleted condition to nitrogen replete (Zienkiewicz et al., 2020). As mentioned above, ATG8 binds to the major LD surface protein LDSP, in which an ATG8 interaction motif (AIM) was detected (Zienkiewicz et al., 2020). Interestingly, one of the major LD surface proteins in *P. tricornutum*, StLDP, also possesses an AIM (Leyland et al., 2020a). This suggests that macrolipophagy might be involved in LD breakdown. Transmission electron microscopy observations showed that in *N. oceanica*, LDs fuse with the vacuole after transition from nitrogen-depleted to nitrogen-replete condition (Zienkiewicz et al., 2020). This strongly suggests that microlipophagy is also involved in LD turnover in *N. oceanica*.

The LD proteome of *Fistulifera solaris* also reveals the presence of clathrin vesicle coating system and of COP1 coatomer (Nonoyama et al., 2019), like that of *P. tricornutum*. Clathrins have been shown to be involved in macrolipophagy (Oku et al., 2017); hence, in *P. tricornutum* and *F. solaris*, LD breakdown may occur by microlipophagy. In addition, both *P. tricornutum* and *F. solaris* LD proteomes contain a HSP70 homolog. Hsp70 family (which includes HSC70) was shown to play a role in LD protein degradation by CMA (Cuervo and Wong, 2014; Kaushik and Cuervo, 2015), and in the removal of clathrin from clathrin-coated vesicles (Barouch et al., 1994; Rapoport et al., 2008), which is an important step for vesicle fusion and cargo delivery.

To test the potential role of lipophagy in LD mobilization in *F. solaris* and *P. tricornutum*, Nonoyama et al. (2019) used inhibitors of membrane trafficking-related proteins and lipophagy (Nonoyama et al., 2019). Chloroquine was used to prevent fusion of LD and autophagosome, VER-155008 was used to bind to HSP70, and Pitstop 2 was used to bind clathrin. All three inhibitors limited LD shrinkage in *F. solaris* and in *P. tricornutum*, supporting the occurrence of lipophagy processes in LD breakdown, with the probable involvement of HSP70 and clathrin. More specifically, these results are coherent with CMA and microlipophagy playing a role in LD breakdown.

Remobilization of stored energy in LD involves the disassembly of TAG molecules into glycerol and free FAs. Glycerol will then be redirected to the glycolysis pathway, while FA will undergo β-oxidation, which in diatoms can occur in the mitochondria and in the peroxisomes (Chauton et al., 2013). It was recently shown that mitochondrial β-oxidation was prominent in diatoms (Jallet et al., 2020). The first step to catabolize TAG is generally performed by TAG lipases. In *Arabidopsis*, AtSDP1 is a TAG lipase that plays a key role in TAG hydrolysis during seed germination (Eastmond, 2006). Homologs of AtSDP1 have been characterized in stramenopiles, named tgl1 in *P. tricornutum* (Barka et al., 2016), and NoTGL1 and NoTGL2 in *N. oceanica* (Nobusawa et al., 2019). *tgl1* KD and *NoTGL1* KO lead to over-accumulation of TAG, corroborating their role in TAG hydrolysis. NoTGL2, on the other hand, was shown to have a more specific role than NoTGL1, with the degradation of 16:1-containing molecular species of TAG (Nobusawa et al., 2019). Many other putative TAG lipases have been predicted in *P. tricornutum*, *N. oceanica*, and *F. solaris* but need to be investigated (Barka et al., 2016; Nomaguchi et al., 2018; Zienkiewicz et al., 2020).

## Concluding Remarks

Secondary endosymbiosis-derived organisms, with their complicated cellular organizations, have been far less studied than simpler photosynthetic eukaryotes, such as the green alga *C. reinhardtii* or the plant *A. thaliana.* These Archaeplastida models contain primary chloroplasts and have been our reference for decades to unravel unique features of the plant kingdom, such as the synthesis of FA within the stroma, or the coordination of two membrane systems for the biosynthesis of membrane glycerolipids, schematically the ER for the bulk of phospholipids and the plastid for glycolipids. Enzymes have been purified, functionally and molecularly characterized, and metabolic pathways reconstructed at the cellular level. The localization of TAG biogenesis at the ER has been discussed in green algae, likely involving the chloroplast. Trafficking of FA and lipid intermediates and FA editing appear as the last challenges we need in order to collectively comprehend lipid metabolism in Archaeplastida. On the basis of knowledge gathered in other model organisms, we elaborated paradigms that need evaluation in stramenopiles. The structure of the secondary plastid is probably one of the major obstacles to transfer knowledge from *C. reinhardtii* or *A. thaliana*, since no such ER–plastid hybrid structure has ever been studied before. Here, we attempted to summarize efforts put recently on stramenopile models, which emerged also as oleaginous strains for possible biotechnological applications. The secondary plastid appears as a pivotal cellular factory for membrane glycerolipid and TAG production, as well as for the biogenesis of the LD, so we need to refine our understanding of the cooperation of the secondary plastid with other organelles. Many biochemical, cell biology, and genetic analyses still need to be performed. Resolving some of the questions we listed in this review is necessary for biotechnological purposes. Exploring this *terra incognita* is definitely a fascinating mission.

## Author Contributions

All authors listed have made a substantial, direct and intellectual contribution to the work, and approved it for publication.

## Conflict of Interest

The authors declare that the research was conducted in the absence of any commercial or financial relationships that could be construed as a potential conflict of interest.

## References

[B1] Abell B. M. Hahn M. Holbrook L. A. Moloney M. M. (2004). Membrane topology and sequence requirements for oil body targeting of oleosin: role of oleosin topology in oil body targeting. *Plant J.* 37 461–470. 10.1111/j.1365-313X.2003.01988.x 14756765

[B2] Abida H. Dolch L.-J. Meï C. Villanova V. Conte M. Block M. A. (2015). Membrane glycerolipid remodeling triggered by nitrogen and phosphorus starvation in *Phaeodactylum tricornutum*. *Plant Physiol.* 167 118–136. 10.1104/pp.114.252395 25489020 PMC4281014

[B3] Adl S. N. Simpson A. G. B. Farmer M. A. Andersen R. A. Anderson R. O. Barta J. R. (2005). The new higher level classification of eukaryotes with emphasis on the taxonomy of protists. *J Eukaryot. Microbiol.* 52 399–451. 10.1111/j.1550-7408.2005.00053.x 16248873

[B4] Alboresi A. Perin G. Vitulo N. Diretto G. Block M. A. Jouhet J. (2016). Light remodels lipid biosynthesis in *Nannochloropsis gaditana* by modulating carbon partitioning between organelles. *Plant Physiol.* 171 2468–2482. 10.1104/pp.16.00599 27325666 PMC4972283

[B5] Alipanah L. Rohloff J. Winge P. Bones A. M. Brembu T. (2015). Whole-cell response to nitrogen deprivation in the diatom *Phaeodactylum tricornutum*. *J. Exp. Bot.* 66 6281–6296. 10.1093/jxb/erv340 26163699 PMC4588885

[B6] Alipanah L. Winge P. Rohloff J. Najafi J. Brembu T. Bones A. M. (2018). Molecular adaptations to phosphorus deprivation and comparison with nitrogen deprivation responses in the diatom *Phaeodactylum tricornutum*. *PLoS One* 13:e0193335. 10.1371/journal.pone.0193335 29474408 PMC5825098

[B7] Aymé L. Arragain S. Canonge M. Baud S. Touati N. Bimai O. (2018). *Arabidopsis thaliana* DGAT3 is a [2Fe-2S] protein involved in TAG biosynthesis. *Sci. Rep.* 8:17254. 10.1038/s41598-018-35545-7 30467384 PMC6250708

[B8] Barka F. Angstenberger M. Ahrendt T. Lorenzen W. Bode H. B. Büchel C. (2016). Identification of a triacylglycerol lipase in the diatom *Phaeodactylum tricornutum*. *Biochim. Biophys. Acta* 1861 239–248. 10.1016/j.bbalip.2015.12.023 26747649

[B9] Barouch W. Prasad K. Greene L. E. Eisenberg E. (1994). ATPase activity associated with the uncoating of clathrin baskets by Hsp70. *J. Biol. Chem.* 269 28563–28568. 7961802

[B10] Bartz R. Li W.-H. Venables B. Zehmer J. K. Roth M. R. Welti R. (2007). Lipidomics reveals that adiposomes store ether lipids and mediate phospholipid traffic,. *J. Lipid Res.* 48 837–847. 10.1194/jlr.M600413-JLR200 17210984

[B11] Ben M’barek K. Ajjaji D. Chorlay A. Vanni S. Forêt L. Thiam A. R. (2017). ER Membrane phospholipids and surface tension control cellular lipid droplet formation. *Dev. Cell* 41 591–604.e7. 10.1016/j.devcel.2017.05.012 28579322

[B12] Benning C. (2009). Mechanisms of lipid transport involved in organelle biogenesis in plant cells. *Annu. Rev. Cell Dev. Biol.* 25 71–91. 10.1146/annurev.cellbio.042308.113414 19572810

[B13] Binns D. Lee S. Hilton C. L. Jiang Q.-X. Goodman J. M. (2010). Seipin is a discrete homooligomer. *Biochemistry* 49 10747–10755. 10.1021/bi1013003 21062080 PMC3086013

[B14] Bohnert M. (2020). New friends for seipin — Implications of seipin partner proteins in the life cycle of lipid droplets. *Semin. Cell Dev. Biol.* 108 24–32. 10.1016/j.semcdb.2020.04.012 32402516

[B15] Boudière L. Botté C. Y. Saidani N. Lajoie M. Marion J. Bréhélin L. (2012). Galvestine-1, a novel chemical probe for the study of the glycerolipid homeostasis system in plant cells. *Mol. BioSyst.* 8:2023. 10.1039/c2mb25067e 22592295

[B16] Boudière L. Michaud M. Petroutsos D. Rébeillé F. Falconet D. Bastien O. (2014). Glycerolipids in photosynthesis: composition, synthesis and trafficking. *Biochim. Biophys. Acta* 1837 470–480. 10.1016/j.bbabio.2013.09.007 24051056

[B17] Boutet E. El Mourabit H. Prot M. Nemani M. Khallouf E. Colard O. (2009). Seipin deficiency alters fatty acid Δ9 desaturation and lipid droplet formation in Berardinelli-Seip congenital lipodystrophy. *Biochimie* 91 796–803. 10.1016/j.biochi.2009.01.011 19278620

[B18] Burki F. Roger A. J. Brown M. W. Simpson A. G. B. (2020). The new tree of eukaryotes. *Trends Ecol. Evol.* 35 43–55. 10.1016/j.tree.2019.08.008 31606140

[B19] Busseni G. Rocha Jimenez Vieira F. Amato A. Pelletier E. Pierella Karlusich J. J. Ferrante M. I. (2019). Meta-omics reveals genetic flexibility of diatom nitrogen transporters in response to environmental changes. *Mol. Biol. Evol.* 36 2522–2535. 10.1093/molbev/msz157 31259367 PMC6805229

[B20] Butler T. Kapoore R. V. Vaidyanathan S. (2020). *Phaeodactylum tricornutum*: a diatom cell factory. *Trends Biotechnol.* 38 606–622. 10.1016/j.tibtech.2019.12.023 31980300

[B21] Cai Y. Goodman J. M. Pyc M. Mullen R. T. Dyer J. M. Chapman K. D. (2015). *Arabidopsis* SEIPIN proteins modulate triacylglycerol accumulation and influence lipid droplet proliferation. *Plant Cell* 27 2616–2636. 10.1105/tpc.15.00588 26362606 PMC4815042

[B22] Cañavate J. P. Armada I. Hachero-Cruzado I. (2017a). Aspects of phosphorus physiology associated with phosphate-induced polar lipid remodelling in marine microalgae. *J. Plant Physiol.* 214 28–38. 10.1016/j.jplph.2017.03.019 28423307

[B23] Cañavate J. P. Armada I. Hachero-Cruzado I. (2017b). Interspecific variability in phosphorus-induced lipid remodelling among marine eukaryotic phytoplankton. *New Phytol.* 213 700–713. 10.1111/nph.14179 27605045

[B24] Castro I. Eisenberg-Bord M. Persiani E. Rochford J. Schuldiner M. Bohnert M. (2019). Promethin is a conserved seipin partner protein. *Cells* 8:268. 10.3390/cells8030268 30901948 PMC6468817

[B25] Cavalier-Smith T. (1981). Eukaryote kingdoms: seven or nine? *Biosystems* 14 461–481. 10.1016/0303-2647(81)90050-2 7337818

[B26] Cavalier-Smith T. (2018). Kingdom Chromista and its eight phyla: a new synthesis emphasising periplastid protein targeting, cytoskeletal and periplastid evolution, and ancient divergences. *Protoplasma* 255 297–357. 10.1007/s00709-017-1147-3 28875267 PMC5756292

[B27] Cenci U. Bhattacharya D. Weber A. P. M. Colleoni C. Subtil A. Ball S. G. (2017). Biotic host–pathogen interactions as major drivers of plastid endosymbiosis. *Trends Plant Sci.* 22 316–328. 10.1016/j.tplants.2016.12.007 28089380

[B28] Cerón Garcí M. C. Fernández Sevilla J. M. Acién Fernández F. G. Molina Grima E. García Camacho F. (2000). Mixotrophic growth of *Phaeodactylum tricornutum* on glycerol: growth rate and fatty acid profile. *J. Appl. Phycol.* 12 239–248. 10.1023/A:1008123000002

[B29] Chapman K. D. Aziz M. Dyer J. M. Mullen R. T. (2019). Mechanisms of lipid droplet biogenesis. *Biochem. J.* 476 1929–1942. 10.1042/BCJ20180021 31289128

[B30] Chauton M. S. Winge P. Brembu T. Vadstein O. Bones A. M. (2013). Gene regulation of carbon fixation, storage, and utilization in the diatom *Phaeodactylum tricornutum* acclimated to light/dark cycles. *Plant Physiol.* 161 1034–1048. 10.1104/pp.112.206177 23209127 PMC3561001

[B31] Chen J. E. Smith A. G. (2012). A look at diacylglycerol acyltransferases (DGATs) in algae. *J. Biotechnol.* 162 28–39. 10.1016/j.jbiotec.2012.05.009 22750092

[B32] Chorlay A. Monticelli L. Veríssimo Ferreira J. Ben M’barek K. Ajjaji D. Wang S. (2019). Membrane asymmetry imposes directionality on lipid droplet emergence from the ER. *Dev. Cell* 50 25–42.e7. 10.1016/j.devcel.2019.05.003 31155466

[B33] Chorlay A. Thiam A. R. (2018). An asymmetry in monolayer tension regulates lipid droplet budding direction. *Biophys. J.* 114 631–640. 10.1016/j.bpj.2017.12.014 29414709 PMC5985028

[B34] Choudhary V. El Atab O. Mizzon G. Prinz W. A. Schneiter R. (2020). Seipin and Nem1 establish discrete ER subdomains to initiate yeast lipid droplet biogenesis. *J. Cell Biol.* 219:e201910177. 10.1083/jcb.201910177 32349126 PMC7337503

[B35] Choudhary V. Golani G. Joshi A. S. Cottier S. Schneiter R. Prinz W. A. (2018). Architecture of lipid droplets in endoplasmic reticulum is determined by phospholipid intrinsic curvature. *Curr. Biol.* 28 915–926.e9. 10.1016/j.cub.2018.02.020 29526591 PMC5889118

[B36] Choudhary V. Ojha N. Golden A. Prinz W. A. (2015). A conserved family of proteins facilitates nascent lipid droplet budding from the ER. *J. Cell Biol.* 211 261–271. 10.1083/jcb.201505067 26504167 PMC4621845

[B37] Chung J. Wu X. Lambert T. J. Lai Z. W. Walther T. C. Farese R. V. (2019). LDAF1 and Seipin form a lipid droplet assembly complex. *Dev. Cell* 51 551.e–563.e. 10.1016/j.devcel.2019.10.006 31708432 PMC7235935

[B38] Conte M. Lupette J. Seddiki K. Meï C. Dolch L.-J. Gros V. (2018). Screening for biologically annotated drugs that trigger triacylglycerol accumulation in the diatom *Phaeodactylum*. *Plant Physiol.* 177 532–552. 10.1104/pp.17.01804 29535162 PMC6001342

[B39] Corteggiani Carpinelli E. Telatin A. Vitulo N. Forcato C. D’Angelo M. Schiavon R. (2014). Chromosome scale genome assembly and transcriptome profiling of *Nannochloropsis gaditana* in nitrogen depletion. *Mol. Plant* 7 323–335. 10.1093/mp/sst120 23966634

[B40] Cruz de Carvalho M. H. Sun H.-X. Bowler C. Chua N.-H. (2016). Noncoding and coding transcriptome responses of a marine diatom to phosphate fluctuations. *New Phytol.* 210 497–510. 10.1111/nph.13787 26680538

[B41] Cuervo A. M. Wong E. (2014). Chaperone-mediated autophagy: roles in disease and aging. *Cell Res.* 24 92–104. 10.1038/cr.2013.153 24281265 PMC3879702

[B42] Cui Y. Zhao J. Wang Y. Qin S. Lu Y. (2018). Characterization and engineering of a dual-function diacylglycerol acyltransferase in the oleaginous marine diatom *Phaeodactylum tricornutum*. *Biotechnol. Biofuels* 11:32. 10.1186/s13068-018-1029-8 29449880 PMC5806285

[B43] Cui Y. Zheng G. Li X. Lin H. Jiang P. Qin S. (2013). Cloning and characterization of a novel diacylglycerol acyltransferase from the diatom *Phaeodactylum tricornutum*. *J. Appl. Phycol.* 25 1509–1512. 10.1007/s10811-013-9991-9

[B44] Curtis B. A. Tanifuji G. Burki F. Gruber A. Irimia M. Maruyama S. (2012). Algal genomes reveal evolutionary mosaicism and the fate of nucleomorphs. *Nature* 492 59–65. 10.1038/nature11681 23201678

[B45] Dahlqvist A. Stahl U. Lenman M. Banas A. Lee M. Sandager L. (2000). Phospholipid:diacylglycerol acyltransferase: an enzyme that catalyzes the acyl-CoA-independent formation of triacylglycerol in yeast and plants. *Proc. Natl. Acad. Sci. U.S.A.* 97 6487–6492. 10.1073/pnas.120067297 10829075 PMC18631

[B46] Dellero Y. Cagnac O. Rose S. Seddiki K. Cussac M. Morabito C. (2018a). Proposal of a new thraustochytrid genus *Hondaea* gen. nov. and comparison of its lipid dynamics with the closely related pseudo-cryptic genus *Aurantiochytrium*. *Algal Res.* 35 125–141. 10.1016/j.algal.2018.08.018

[B47] Dellero Y. Rose S. Metton C. Morabito C. Lupette J. Jouhet J. (2018b). Ecophysiology and lipid dynamics of a eukaryotic mangrove decomposer: autecology and ecophysiology of *Aurantiochytrium limacinum*. *Environ. Microbiol.* 20 3057–3068. 10.1111/1462-2920.14346 29968288

[B48] Derelle R. López-García P. Timpano H. Moreira D. (2016). A phylogenomic framework to study the diversity and evolution of Stramenopiles (=Heterokonts). *Mol. Biol. Evol.* 33 2890–2898. 10.1093/molbev/msw168 27512113 PMC5482393

[B49] Dolch L.-J. Lupette J. Tourcier G. Bedhomme M. Collin S. Magneschi L. (2017a). Nitric oxide mediates nitrite-sensing and acclimation and triggers a remodeling of lipids. *Plant Physiol.* 175 1407–1423. 10.1104/pp.17.01042 28924015 PMC5664477

[B50] Dolch L.-J. Maréchal E. (2015). Inventory of fatty acid desaturases in the pennate diatom *Phaeodactylum tricornutum*. *Mar. Drugs* 13 1317–1339. 10.3390/md13031317 25786062 PMC4377986

[B51] Dolch L.-J. Rak C. Perin G. Tourcier G. Broughton R. Leterrier M. (2017b). A palmitic acid elongase affects eicosapentaenoic acid and plastidial monogalactosyldiacylglycerol levels in *Nannochloropsis*. *Plant Physiol.* 173 742–759. 10.1104/pp.16.01420 27895203 PMC5210741

[B52] Dorell R. G. Gile G. McCallum G. Méheust R. Bapteste E. P. Klinger C. M. (2017). Chimeric origins of ochrophytes and haptophytes revealed through an ancient plastid proteome. *eLife* 6:e23717. 10.7554/eLife.23717 28498102 PMC5462543

[B53] Eastmond P. J. (2006). *SUGAR-DEPENDENT1* encodes a patatin domain triacylglycerol lipase that initiates storage oil breakdown in germinating *Arabidopsis* Seeds. *Plant Cell* 18 665–675. 10.1105/tpc.105.040543 16473965 PMC1383641

[B54] Eisenberg-Bord M. Mari M. Weill U. Rosenfeld-Gur E. Moldavski O. Castro I. G. (2018). Identification of seipin-linked factors that act as determinants of a lipid droplet subpopulation. *J. Cell Biol.* 217 269–282. 10.1083/jcb.201704122 29187527 PMC5748981

[B55] Fábregas J. Maseda A. Domínguez A. Ferreira M. Otero A. (2002). Changes in the cell composition of the marine microalga, *Nannochloropsis gaditana*, during a light:dark cycle. *Biotechnol. Lett.* 24 1699–1703. 10.1023/A:1020661719272

[B56] Falarz L. J. Xu Y. Caldo K. M. P. Garroway C. J. Singer S. D. Chen G. (2020). Characterization of the diversification of phospholipid:diacylglycerol acyltransferases in the green lineage. *Plant J.* 103 2025–2038. 10.1111/tpj.14880 32538516

[B57] Fan J. Andre C. Xu C. (2011). A chloroplast pathway for the de novo biosynthesis of triacylglycerol in *Chlamydomonas reinhardtii*. *FEBS Lett.* 585 1985–1991. 10.1016/j.febslet.2011.05.018 21575636

[B58] Fan X. Qiu H. Han W. Wang Y. Xu D. Zhang X. (2020). Phytoplankton pangenome reveals extensive prokaryotic horizontal gene transfer of diverse functions. *Sci. Adv.* 6:eaba0111. 10.1126/sciadv.aba0111 32494685 PMC7190310

[B59] Fang X. Wei C. Zhao-Ling C. Fan O. (2004). Effects of organic carbon sources on cell growth and eicosapentaenoic acid content of *Nannochloropsis* sp. *J. Appl. Phycol.* 16 499–503. 10.1007/s10811-004-5520-1

[B60] Fawley M. W. Jameson I. Fawley K. P. (2015). The phylogeny of the genus *Nannochloropsis* (Monodopsidaceae, Eustigmatophyceae), with descriptions of *N. australis* sp. nov. and *Microchloropsis* gen. nov. *Phycologia* 54 545–552. 10.2216/15-60.1

[B61] Flori S. Jouneau P.-H. Finazzi G. Maréchal E. Falconet D. (2016). Ultrastructure of the periplastidial compartment of the diatom *Phaeodactylum tricornutum*. *Protist* 167 254–267. 10.1016/j.protis.2016.04.001 27179349

[B62] Fossier Marchan L. Lee Chang K. J. Nichols P. D. Mitchell W. J. Polglase J. L. Gutierrez T. (2018). Taxonomy, ecology and biotechnological applications of thraustochytrids: a review. *Biotechnol. Adv.* 36 26–46. 10.1016/j.biotechadv.2017.09.003 28911809

[B63] Franz A. K. Danielewicz M. A. Wong D. M. Anderson L. A. Boothe J. R. (2013). Phenotypic screening with oleaginous microalgae reveals modulators of lipid productivity. *ACS Chem. Biol.* 8 1053–1062. 10.1021/cb300573r 23521767

[B64] Fucikova K. Lewis L. A. (2012). Intersection of *Chlorella*, *Muriella* and *Bracteacoccus*: resurrecting the genus *Chromochloris* Kol et Chodat (Chlorophyceae, Chlorophyta). *Fottea* 12 83–93. 10.5507/fot.2012.007

[B65] Gao Z. Meng C. Gao H. Zhang X. Xu D. Su Y. (2013). Analysis of mRNA expression profiles of carotenogenesis and astaxanthin production of *Haematococcus pluvialis* under exogenous 2, 4-epibrassinolide (EBR). *Biol. Res.* 46 201–206. 10.4067/S0716-97602013000200012 23959019

[B66] Gill S. Willette S. Dungan B. Jarvis J. Schaub T. VanLeeuwen D. (2018). Suboptimal temperature acclimation affects kennedy pathway gene expression, lipidome and metabolite profile of *Nannochloropsis salina* during PUFA enriched TAG synthesis. *Mar. Drugs* 16:425. 10.3390/md16110425 30388843 PMC6266265

[B67] Gilson P. R. Su V. Slamovits C. H. Reith M. E. Keeling P. J. McFadden G. I. (2006). Complete nucleotide sequence of the chlorarachniophyte nucleomorph: nature’s smallest nucleus. *Proc. Natl. Acad. Sci. U.S.A.* 103 9566–9571. 10.1073/pnas.0600707103 16760254 PMC1480447

[B68] Goncalves E. C. Wilkie A. C. Kirst M. Rathinasabapathi B. (2016). Metabolic regulation of triacylglycerol accumulation in the green algae: identification of potential targets for engineering to improve oil yield. *Plant Biotechnol. J.* 14 1649–1660. 10.1111/pbi.12523 26801206 PMC5066758

[B69] Gong Y. Zhang J. Guo X. Wan X. Liang Z. Hu C. J. (2013). Identification and characterization of PtDGAT2B, an acyltransferase of the DGAT2 acyl-Coenzyme A: diacylglycerol acyltransferase family in the diatom *Phaeodactylum tricornutum*. *FEBS Lett.* 587 481–487. 10.1016/j.febslet.2013.01.015 23337871

[B70] Goodson C. Roth R. Wang Z. T. Goodenough U. (2011). Structural correlates of cytoplasmic and chloroplast lipid body synthesis in chlamydomonas reinhardtii and stimulation of lipid body production with acetate boost. *Eukaryot. Cell* 10 1592–1606. 10.1128/EC.05242-11 22037181 PMC3232719

[B71] Gould S. B. Maier U.-G. Martin W. F. (2015). Protein import and the origin of red complex plastids. *Curr. Biol.* 25 R515–R521. 10.1016/j.cub.2015.04.033 26079086

[B72] Greer M. S. Cai Y. Gidda S. K. Esnay N. Kretzschmar F. K. Seay D. (2020). SEIPIN isoforms interact with the membrane-tethering protein VAP27-1 for lipid droplet formation. *Plant Cell* 32 2932–2950. 10.1105/tpc.19.00771 32690719 PMC7474298

[B73] Grippa A. Buxó L. Mora G. Funaya C. Idrissi F.-Z. Mancuso F. (2015). The seipin complex Fld1/Ldb16 stabilizes ER–lipid droplet contact sites. *J. Cell Biol.* 211 829–844. 10.1083/jcb.201502070 26572621 PMC4657162

[B74] Grosche C. Hempel F. Bolte K. Zauner S. Maier U. G. (2014). The periplastidal compartment: a naturally minimized eukaryotic cytoplasm. *Curr. Opin. Microbiol.* 22 88–93. 10.1016/j.mib.2014.09.017 25460801

[B75] Guihéneuf F. Leu S. Zarka A. Khozin-Goldberg I. Khalilov I. Boussiba S. (2011). Cloning and molecular characterization of a novel acyl-CoA:diacylglycerol acyltransferase 1-like gene (PtDGAT1) from the diatom *Phaeodactylum tricornutum*: *P. tricornutum* DGAT1 cloning and characterization. *FEBS J.* 278 3651–3666. 10.1111/j.1742-4658.2011.08284.x 21812932

[B76] Haslam R. P. Hamilton M. L. Economou C. K. Smith R. Hassall K. L. Napier J. A. (2020). Overexpression of an endogenous type 2 diacylglycerol acyltransferase in the marine diatom *Phaeodactylum tricornutum* enhances lipid production and omega-3 long-chain polyunsaturated fatty acid content. *Biotechnol. Biofuels* 13:87. 10.1186/s13068-020-01726-8 32467729 PMC7227059

[B77] Hernández M. L. Whitehead L. He Z. Gazda V. Gilday A. Kozhevnikova E. (2012). A cytosolic acyltransferase contributes to triacylglycerol synthesis in sucrose-rescued arabidopsis seed oil catabolism mutants. *Plant Physiol.* 160 215–225. 10.1104/pp.112.201541 22760209 PMC3440200

[B78] Hu Q. Sommerfeld M. Jarvis E. Ghirardi M. Posewitz M. Seibert M. (2008). Microalgal triacylglycerols as feedstocks for biofuel production: perspectives and advances. *Plant J.* 54 621–639. 10.1111/j.1365-313X.2008.03492.x 18476868

[B79] Hulatt C. J. Smolina I. Dowle A. Kopp M. Vasanth G. K. Hoarau G. G. (2020). Proteomic and transcriptomic patterns during lipid remodeling in *Nannochloropsis gaditana*. *IJMS* 21:6946. 10.3390/ijms21186946 32971781 PMC7554720

[B80] Ischebeck T. Krawczyk H. E. Mullen R. T. Dyer J. M. Chapman K. D. (2020). Lipid droplets in plants and algae: distribution, formation, turnover and function. *Semin. Cell Dev. Biol.* 108 82–93. 10.1016/j.semcdb.2020.02.014 32147380

[B81] Iwai M. Hori K. Sasaki-Sekimoto Y. Shimojima M. Ohta H. (2015). Manipulation of oil synthesis in *Nannochloropsis* strain NIES-2145 with a phosphorus starvation–inducible promoter from *Chlamydomonas reinhardtii*. *Front. Microbiol.* 6:912. 10.3389/fmicb.2015.00912 26441858 PMC4561341

[B82] Jackson C. J. Gornik S. G. Waller R. F. (2012). A tertiary plastid gains RNA editing in its new host. *Mol. Biol. Evol.* 30 788–792. 10.1093/molbev/mss270 23197592

[B83] Jallet D. Xing D. Hughes A. Moosburner M. Simmons M. P. Allen A. E. (2020). Mitochondrial fatty acid β−oxidation is required for storage-lipid catabolism in a marine diatom. *New Phytol.* 228 946–958. 10.1111/nph.16744 32535932

[B84] Janssen J. H. Driessen J. L. S. P. Lamers P. P. Wijffels R. H. Barbosa M. J. (2018). Effect of initial biomass-specific photon supply rate on fatty acid accumulation in nitrogen depleted *Nannochloropsis gaditana* under simulated outdoor light conditions. *Algal Res.* 35 595–601. 10.1016/j.algal.2018.10.002

[B85] Janssen J. H. Lamers P. P. de Vos R. C. H. Wijffels R. H. Barbosa M. J. (2019a). Translocation and de novo synthesis of eicosapentaenoic acid (EPA) during nitrogen starvation in *Nannochloropsis gaditana*. *Algal Res.* 37 138–144. 10.1016/j.algal.2018.11.025

[B86] Janssen J. H. Spoelder J. Koehorst J. J. Schaap P. J. Wijffels R. H. Barbosa M. J. (2020). Time-dependent transcriptome profile of genes involved in triacylglycerol (TAG) and polyunsaturated fatty acid synthesis in *Nannochloropsis gaditana* during nitrogen starvation. *J. Appl. Phycol.* 32 1153–1164. 10.1007/s10811-019-02021-2

[B87] Janssen J. H. Wijffels R. H. Barbosa M. J. (2019b). Lipid production in *Nannochloropsis gaditana* during nitrogen starvation. *Biology* 8:5. 10.3390/biology8010005 30626148 PMC6466408

[B88] Jaussaud A. Lupette J. Salvaing J. Jouhet J. Bastien O. Gromova M. (2020). Stepwise biogenesis of subpopulations of lipid droplets in nitrogen starved *Phaeodactylum tricornutum* cells. *Front. Plant Sci.* 11:48. 10.3389/fpls.2020.00048 32117386 PMC7026457

[B89] Jensen P. E. Leister D. (2014). Chloroplast evolution, structure and functions. *F1000Prime Rep.* 6:40. 10.12703/P6-40 24991417 PMC4075315

[B90] Jia J. Han D. Gerken H. G. Li Y. Sommerfeld M. Hu Q. (2015). Molecular mechanisms for photosynthetic carbon partitioning into storage neutral lipids in *Nannochloropsis oceanica* under nitrogen-depletion conditions. *Algal Res.* 7 66–77. 10.1016/j.algal.2014.11.005

[B91] Joshi A. S. Nebenfuehr B. Choudhary V. Satpute-Krishnan P. Levine T. P. Golden A. (2018). Lipid droplet and peroxisome biogenesis occur at the same ER subdomains. *Nat. Commun.* 9:2940. 10.1038/s41467-018-05277-3 30054481 PMC6063926

[B92] Jürgens G. (2004). Membrane trafficking in plants. *Annu. Rev. Cell Dev. Biol.* 20 481–504. 10.1146/annurev.cellbio.20.082503.103057 15473849

[B93] Kaushik S. Cuervo A. M. (2015). Degradation of lipid droplet-associated proteins by chaperone-mediated autophagy facilitates lipolysis. *Nat. Cell Biol.* 17 759–770. 10.1038/ncb3166 25961502 PMC4449813

[B94] Keeling P. J. (2010). The endosymbiotic origin, diversification and fate of plastids. *Phil. Trans. R. Soc. Lond. B* 365 729–748. 10.1098/rstb.2009.0103 20124341 PMC2817223

[B95] Keeling P. J. (2013). The number, speed, and impact of plastid endosymbioses in eukaryotic evolution. *Annu. Rev. Plant Biol.* 64 583–607. 10.1146/annurev-arplant-050312-120144 23451781

[B96] Kennedy E. P. Weiss S. B. (1956). The function of cytidine coenzymes in the biosynthesis of phospholipides. *J. Biol. Chem.* 222 193–214. 10.1016/S0021-9258(19)50785-2 13366993

[B97] Kilian O. Kroth P. G. (2004). Identification and characterization of a new conserved motif within the presequence of proteins targeted into complex diatom plastids: protein targeting into diatom plastids. *Plant J.* 41 175–183. 10.1111/j.1365-313X.2004.02294.x 15634195

[B98] Kory N. Farese R. V. Walther T. C. (2016). Targeting fat: mechanisms of protein localization to lipid droplets. *Trends Cell Biol.* 26 535–546. 10.1016/j.tcb.2016.02.007 26995697 PMC4976449

[B99] Leyland B. Boussiba S. Khozin-Goldberg I. (2020a). A review of diatom lipid droplets. *Biology* 9:38. 10.3390/biology9020038 32098118 PMC7168155

[B100] Leyland B. Zarka A. Didi-Cohen S. Boussiba S. Khozin-Goldberg I. (2020b). High resolution proteome of lipid droplets isolated from the pennate diatom *Phaeodactylum tricornutum* (Bacillariophyceae) strain pt4 provides mechanistic insights into complex intracellular coordination during nitrogen deprivation. *J. Phycol.* 56 1642–1663. 10.1111/jpy.13063 32779202

[B101] Li D.-W. Cen S.-Y. Liu Y.-H. Balamurugan S. Zheng X.-Y. Alimujiang A. (2016). A type 2 diacylglycerol acyltransferase accelerates the triacylglycerol biosynthesis in heterokont oleaginous microalga *Nannochloropsis oceanica*. *J. Biotechnol.* 229 65–71. 10.1016/j.jbiotec.2016.05.005 27164260

[B102] Li J. Han D. Wang D. Ning K. Jia J. Wei L. (2014). Choreography of transcriptomes and lipidomes of *Nannochloropsis* reveals the mechanisms of oil synthesis in microalgae. *Plant Cell* 26 1645–1665. 10.1105/tpc.113.121418 24692423 PMC4036577

[B103] Li L. Wang S. Wang H. Sahu S. K. Marin B. Li H. (2020). The genome of *Prasinoderma coloniale* unveils the existence of a third phylum within green plants. *Nat. Ecol. Evol.* 4 1220–1231. 10.1038/s41559-020-1221-7 32572216 PMC7455551

[B104] Liang J. Iqbal S. Wen F. Tong M. Liu J. (2019a). Phosphorus-induced lipid class alteration revealed by lipidomic and transcriptomic profiling in oleaginous microalga *Nannochloropsis* sp. PJ12. *Mar. Drugs* 17:519. 10.3390/md17090519 31484443 PMC6780086

[B105] Liang J. Wen F. Liu J. (2019b). Transcriptomic and lipidomic analysis of an EPA-containing *Nannochloropsis* sp. PJ12 in response to nitrogen deprivation. *Sci. Rep.* 9:4540. 10.1038/s41598-019-41169-2 30872742 PMC6418175

[B106] Li-Beisson Y. Shorrosh B. Beisson F. Andersson M. X. Arondel V. Bates P. D. (2010). Acyl-lipid metabolism. *Arabidopsis Book* 8:e0133. 10.1199/tab.0133 22303259 PMC3244904

[B107] Li-Beisson Y. Thelen J. J. Fedosejevs E. Harwood J. L. (2019). The lipid biochemistry of eukaryotic algae. *Prog. Lipid Res.* 74 31–68. 10.1016/j.plipres.2019.01.003 30703388

[B108] Lu Y. Wang X. Balamurugan S. Yang W.-D. Liu J.-S. Dong H.-P. (2017). Identification of a putative seipin ortholog involved in lipid accumulation in marine microalga *Phaeodactylum tricornutum*. *J. Appl. Phycol.* 29 2821–2829. 10.1007/s10811-017-1173-8

[B109] Lupette J. Benning C. (2020). Human health benefits of very-long-chain polyunsaturated fatty acids from microalgae. *Biochimie* 178 15–25. 10.1016/j.biochi.2020.04.022 32389760

[B110] Lupette J. Jaussaud A. Seddiki K. Morabito C. Brugière S. Schaller H. (2019). The architecture of lipid droplets in the diatom *Phaeodactylum tricornutum*. *Algal Res.* 38:101415. 10.1016/j.algal.2019.101415

[B111] Lupette J. Maréchal E. (2018). “Phytoplankton glycerolipids: challenging but promising prospects from biomedicine to green chemistry and biofuels,” in *Blue Biotechnology*, eds La Barre S. Bates S. S. (Weinheim: Wiley-VCH), 191–215. 10.1002/9783527801718.ch6

[B112] Ma X.-N. Chen T.-P. Yang B. Liu J. Chen F. (2016). Lipid production from *Nannochloropsis*. *Mar. Drugs* 14:61. 10.3390/md14040061 27023568 PMC4849066

[B113] Maeda Y. Sunaga Y. Yoshino T. Tanaka T. (2014). Oleosome-associated protein of the oleaginous diatom *Fistulifera solaris* contains an endoplasmic reticulum-targeting signal sequence. *Mar. Drugs* 12 3892–3903. 10.3390/md12073892 24983635 PMC4113804

[B114] Magré J. Delépine M. Khallouf E. Gedde-Dahl T. Van Maldergem L. Sobel E. (2001). Identification of the gene altered in Berardinelli–Seip congenital lipodystrophy on chromosome 11q13. *Nat. Genet.* 28 365–370. 10.1038/ng585 11479539

[B115] Maier U. G. Hofmann C. J. Eschbach S. Wolters J. Igloi G. L. (1991). Demonstration of nucleomorph-encoded eukaryotic small subunit ribosomal RNA in cryptomonads. *Mol. Gen. Genet.* 230 155–160. 10.1007/BF00290663 1720859

[B116] Marechal E. (2018). Primary endosymbiosis: emergence of the primary chloroplast and the chromatophore, two independent events. *Methods Mol. Biol.* 1829, 3–16. 10.1007/978-1-4939-8654-5_1 29987711

[B117] Mayers J. J. Flynn K. J. Shields R. J. (2014). Influence of the N:P supply ratio on biomass productivity and time-resolved changes in elemental and bulk biochemical composition of *Nannochloropsis* sp. *Bioresour. Technol.* 169 588–595. 10.1016/j.biortech.2014.07.048 25103036

[B118] McFie P. J. Stone S. L. Banman S. L. Stone S. J. (2010). Topological orientation of Acyl-CoA:Diacylglycerol Acyltransferase-1 (DGAT1) and identification of a putative active site histidine and the role of the n terminus in dimer/tetramer formation. *J. Biol. Chem.* 285 37377–37387. 10.1074/jbc.M110.163691 20876538 PMC2988343

[B119] Menegol T. Romero-Villegas G. I. López-Rodríguez M. Navarro-López E. López-Rosales L. Chisti Y. (2019). Mixotrophic production of polyunsaturated fatty acids and carotenoids by the microalga *Nannochloropsis gaditana*. *J. Appl. Phycol.* 31 2823–2832. 10.1007/s10811-019-01828-3

[B120] Meng Y. Cao X. Yang M. Liu J. Yao C. Xue S. (2019). Glycerolipid remodeling triggered by phosphorous starvation and recovery in *Nannochloropsis oceanica*. *Algal Res.* 39:101451. 10.1016/j.algal.2019.101451

[B121] Meng Y. Cao X. Yao C. Xue S. Yang Q. (2017). Identification of the role of polar glycerolipids in lipid metabolism and their acyl attribution for TAG accumulation in *Nannochloropsis oceanica*. *Algal Res.* 24 122–129. 10.1016/j.algal.2017.03.004

[B122] Meng Y. Jiang J. Wang H. Cao X. Xue S. Yang Q. (2015). The characteristics of TAG and EPA accumulation in *Nannochloropsis oceanica* IMET1 under different nitrogen supply regimes. *Bioresour. Technol.* 179 483–489. 10.1016/j.biortech.2014.12.012 25575208

[B123] Morabito C. Aiese Cigliano R. Maréchal E. Rébeillé F. Amato A. (2020). Illumina and PacBio DNA sequencing data, de novo assembly and annotation of the genome of *Aurantiochytrium limacinum* strain CCAP_4062/1. *Data Brief* 31:105729. 10.1016/j.dib.2020.105729 32490088 PMC7262427

[B124] Mühlroth A. Li K. Røkke G. Winge P. Olsen Y. Hohmann-Marriott M. (2013). Pathways of lipid metabolism in marine algae, co-expression network, bottlenecks and candidate genes for enhanced production of EPA and DHA in species of chromista. *Mar. Drugs* 11 4662–4697. 10.3390/md11114662 24284429 PMC3853752

[B125] Mühlroth A. Winge P. El Assimi A. Jouhet J. Maréchal E. Hohmann-Marriott M. F. (2017). Mechanisms of phosphorus acquisition and lipid class remodeling under P limitation in a marine microalga. *Plant Physiol.* 175 1543–1559. 10.1104/pp.17.00621 29051196 PMC5717724

[B126] Munz J. Xiong Y. Kim J. Y. H. Sung Y. J. Seo S. Hong R. H. (2020). Arginine-fed cultures generates triacylglycerol by triggering nitrogen starvation responses during robust growth in *Chlamydomonas*. *Algal Res.* 46:101782. 10.1016/j.algal.2019.101782

[B127] Murakami R. Hashimoto H. (2009). Unusual nuclear division in *Nannochloropsis oculata* (Eustigmatophyceae, Heterokonta) which may ensure faithful transmission of secondary plastids. *Protist* 160 41–49. 10.1016/j.protis.2008.09.002 19013102

[B128] Mus F. Toussaint J.-P. Cooksey K. E. Fields M. W. Gerlach R. Peyton B. M. (2013). Physiological and molecular analysis of carbon source supplementation and pH stress-induced lipid accumulation in the marine diatom *Phaeodactylum tricornutum*. *Appl. Microbiol. Biotechnol.* 97 3625–3642. 10.1007/s00253-013-4747-7 23463245

[B129] Nakamura Y. (2013). Phosphate starvation and membrane lipid remodeling in seed plants. *Prog. Lipid Res.* 52 43–50. 10.1016/j.plipres.2012.07.002 22954597

[B130] Nettebrock N. T. Bohnert M. (2020). Born this way – Biogenesis of lipid droplets from specialized ER subdomains. *Biochim. Biophys. Acta* 1865:158448. 10.1016/j.bbalip.2019.04.008 31028912

[B131] Nobusawa T. Hori K. Mori H. Kurokawa K. Ohta H. (2017). Differently localized lysophosphatidic acid acyltransferases crucial for triacylglycerol biosynthesis in the oleaginous alga *Nannochloropsis*. *Plant J.* 90 547–559. 10.1111/tpj.13512 28218992

[B132] Nobusawa T. Yamakawa-Ayukawa K. Saito F. Nomura S. Takami A. Ohta H. (2019). A homolog of *Arabidopsis* SDP1 lipase in *Nannochloropsis* is involved in degradation of de novo-synthesized triacylglycerols in the endoplasmic reticulum. *Biochim. Biophys. Acta* 1864 1185–1193. 10.1016/j.bbalip.2019.05.013 31152796

[B133] Nojima D. Yoshino T. Maeda Y. Tanaka M. Nemoto M. Tanaka T. (2013). Proteomics analysis of oil body-associated proteins in the oleaginous diatom. *J. Proteome Res.* 12 5293–5301. 10.1021/pr4004085 23879348

[B134] Nomaguchi T. Maeda Y. Liang Y. Yoshino T. Asahi T. Tanaka T. (2018). Comprehensive analysis of triacylglycerol lipases in the oleaginous diatom *Fistulifera solaris* JPCC DA0580 with transcriptomics under lipid degradation. *J. Biosci. Bioeng.* 126 258–265. 10.1016/j.jbiosc.2018.03.003 29628268

[B135] Nonoyama T. Nojima D. Maeda Y. Noda M. Yoshino T. Matsumoto M. (2019). Proteomics analysis of lipid droplets indicates involvement of membrane trafficking proteins in lipid droplet breakdown in the oleaginous diatom *Fistulifera solaris*. *Algal Res.* 44:101660. 10.1016/j.algal.2019.101660

[B136] Oborník M. (2019). Endosymbiotic evolution of algae, secondary heterotrophy and parasitism. *Biomolecules* 9:266. 10.3390/biom9070266 31288476 PMC6681372

[B137] Oku M. Maeda Y. Kagohashi Y. Kondo T. Yamada M. Fujimoto T. (2017). Evidence for ESCRT- and clathrin-dependent microautophagy. *J. Cell Biol.* 216 3263–3274. 10.1083/jcb.201611029 28838958 PMC5626533

[B138] Osuna-Cruz C. M. Bilcke G. Vancaester E. De Decker S. Bones A. M. Winge P. (2020). The *Seminavis robusta* genome provides insights into the evolutionary adaptations of benthic diatoms. *Nat. Commun.* 11:3320. 10.1038/s41467-020-17191-8 32620776 PMC7335047

[B139] Pagac M. Cooper D. E. Qi Y. Lukmantara I. E. Mak H. Y. Wu Z. (2016). SEIPIN regulates lipid droplet expansion and adipocyte development by modulating the activity of Glycerol-3-phosphate Acyltransferase. *Cell Rep.* 17 1546–1559. 10.1016/j.celrep.2016.10.037 27806294 PMC5647143

[B140] Parks M. B. Nakov T. Ruck E. C. Wickett N. J. Alverson A. J. (2018). Phylogenomics reveals an extensive history of genome duplication in diatoms (Bacillariophyta). *Am. J. Bot.* 105 330–347. 10.1002/ajb2.1056 29665021

[B141] Pasquet V. Ulmann L. Mimouni V. Guihéneuf F. Jacquette B. Morant-Manceau A. (2014). Fatty acids profile and temperature in the cultured marine diatom *Odontella aurita*. *J. Appl. Phycol.* 26 2265–2271. 10.1007/s10811-014-0252-3

[B142] Peled E. Leu S. Zarka A. Weiss M. Pick U. Khozin-Goldberg I. (2011). Isolation of a novel oil globule protein from the green alga *Haematococcus pluvialis* (Chlorophyceae). *Lipids* 46 851–861. 10.1007/s11745-011-3579-4 21732215

[B143] Petroutsos D. Amiar S. Abida H. Dolch L.-J. Bastien O. Rébeillé F. (2014). Evolution of galactoglycerolipid biosynthetic pathways – From cyanobacteria to primary plastids and from primary to secondary plastids. *Prog. Lipid Res.* 54 68–85. 10.1016/j.plipres.2014.02.001 24594266

[B144] Poliner E. Panchy N. Newton L. Wu G. Lapinsky A. Bullard B. (2015). Transcriptional coordination of physiological responses in *Nannochloropsis oceanica* CCMP1779 under light/dark cycles. *Plant J.* 83 1097–1113. 10.1111/tpj.12944 26216534

[B145] Radakovits R. Jinkerson R. E. Fuerstenberg S. I. Tae H. Settlage R. E. Boore J. L. (2012). Draft genome sequence and genetic transformation of the oleaginous alga *Nannochloropsis gaditana*. *Nat. Commun.* 3:686. 10.1038/ncomms1688 22353717 PMC3293424

[B146] Rapoport I. Boll W. Yu A. Böcking T. Kirchhausen T. (2008). A motif in the clathrin heavy chain required for the Hsc70/Auxilin uncoating reaction. *MBoC* 19 405–413. 10.1091/mbc.e07-09-0870 17978091 PMC2174180

[B147] Remmers I. M. D’Adamo S. Martens D. E. de Vos R. C. H. Mumm R. America A. H. P. (2018). Orchestration of transcriptome, proteome and metabolome in the diatom *Phaeodactylum tricornutum* during nitrogen limitation. *Algal Res.* 35 33–49. 10.1016/j.algal.2018.08.012

[B148] Renne M. F. Klug Y. A. Carvalho P. (2020). Lipid droplet biogenesis: a mystery “unmixing”? *Semin. Cell Dev. Biol.* 108 14–23. 10.1016/j.semcdb.2020.03.001 32192830

[B149] Reyes-Prieto A. Weber A. P. M. Bhattacharya D. (2007). The origin and establishment of the plastid in algae and plants. *Annu. Rev. Genet.* 41 147–168. 10.1146/annurev.genet.41.110306.130134 17600460

[B150] Roughan P. G. Slack C. R. (1982). Cellular organization of glycerolipid metabolism. *Annu. Rev. Plant. Physiol.* 33 97–132. 10.1146/annurev.pp.33.060182.000525

[B151] Saha S. Enugutti B. Rajakumari S. Rajasekharan R. (2006). Cytosolic triacylglycerol biosynthetic pathway in oilseeds. molecular cloning and expression of peanut cytosolic Diacylglycerol Acyltransferase. *Plant Physiol.* 141 1533–1543. 10.1104/pp.106.082198 16798944 PMC1533943

[B152] Salo V. T. Belevich I. Li S. Karhinen L. Vihinen H. Vigouroux C. (2016). Seipin regulates ER –lipid droplet contacts and cargo delivery. *EMBO J.* 35 2699–2716. 10.15252/embj.201695170 27879284 PMC5167346

[B153] Salo V. T. Li S. Vihinen H. Hölttä-Vuori M. Szkalisity A. Horvath P. (2019). Seipin facilitates triglyceride flow to lipid droplet and counteracts droplet ripening via endoplasmic reticulum contact. *Dev. Cell* 50 478–493.e9. 10.1016/j.devcel.2019.05.016 31178403

[B154] Santinho A. Salo V. T. Chorlay A. Li S. Zhou X. Omrane M. (2020). Membrane curvature catalyzes lipid droplet assembly. *Curr. Biol.* 30 2481–2494.e6. 10.1016/j.cub.2020.04.066 32442467

[B155] Santucci P. Johansen M. D. Point V. Poncin I. Viljoen A. Cavalier J.-F. (2019). Nitrogen deprivation induces triacylglycerol accumulation, drug tolerance and hypervirulence in mycobacteria. *Sci. Rep.* 9:8667. 10.1038/s41598-019-45164-5 31209261 PMC6572852

[B156] Sato N. Awai K. (2017). “Prokaryotic Pathway” is not prokaryotic: noncyanobacterial origin of the chloroplast lipid biosynthetic pathway revealed by comprehensive phylogenomic analysis. *Genome Biol. Evol.* 9 3162–3178. 10.1093/gbe/evx238 29145606 PMC5716074

[B157] Schneider J. C. Roessler P. (1994). Radiolabeling studies of lipids and fatty acids in *Nannochloropsis* (Eustigmatophyceae), an oleaginous marine alga. *J. Phycol.* 30 594–598. 10.1111/j.0022-3646.1994.00594.x

[B158] Seddiki K. Godart F. Aiese Cigliano R. Sanseverino W. Barakat M. Ortet P. (2018). Sequencing, *de novo* assembly, and annotation of the complete genome of a new Thraustochytrid species, strain CCAP_4062/3. *Genome Announc.* 6 e1335–e17. 10.1128/genomeA.01335-17 29545303 PMC5854782

[B159] Serafini T. Orci L. Amherdt M. Brunner M. Kahn R. A. Rothmant J. E. (1991). ADP-Ribosylation factor is a subunit of the coat of Golgi-derived COP-coated vesicles: a novel role for a GTP-binding protein. *Cell* 67 239–253. 10.1016/0092-8674(91)90176-Y 1680566

[B160] Sim M. F. M. Dennis R. J. Aubry E. M. Ramanathan N. Sembongi H. Saudek V. (2013). The human lipodystrophy protein seipin is an ER membrane adaptor for the adipogenic PA phosphatase lipin 1. *Mol. Metab.* 2 38–46. 10.1016/j.molmet.2012.11.002 24024128 PMC3757660

[B161] Simionato D. Block M. A. La Rocca N. Jouhet J. Maréchal E. Finazzi G. (2013). The response of *Nannochloropsis gaditana* to nitrogen starvation includes *de novo* biosynthesis of triacylglycerols, a decrease of chloroplast galactolipids, and reorganization of the photosynthetic apparatus. *Eukaryot. Cell* 12 665–676. 10.1128/EC.00363-12 23457191 PMC3647774

[B162] Stone S. J. Levin M. C. Farese R. V. (2006). Membrane topology and identification of key functional amino acid residues of murine Acyl-CoA:Diacylglycerol Acyltransferase-2. *J. Biol. Chem.* 281 40273–40282. 10.1074/jbc.M607986200 17035227

[B163] Strassert J. F. H. Jamy M. Mylnikov A. P. Tikhonenkov D. V. Burki F. (2019). New phylogenomic analysis of the enigmatic phylum telonemia further resolves the eukaryote tree of life. *Mol. Biol. Evol.* 36 757–765. 10.1093/molbev/msz012 30668767 PMC6844682

[B164] Su W.-C. Lin Y.-H. Pagac M. Wang C.-W. (2019). Seipin negatively regulates sphingolipid production at the ER–LD contact site. *J. Cell Biol.* 218 3663–3680. 10.1083/jcb.201902072 31594806 PMC6829658

[B165] Sui X. Arlt H. Brock K. P. Lai Z. W. DiMaio F. Marks D. S. (2018). Cryo–electron microscopy structure of the lipid droplet–formation protein seipin. *J. Cell Biol.* 217 4080–4091. 10.1083/jcb.201809067 30327422 PMC6279392

[B166] Sui X. Wang K. Gluchowski N. L. Elliott S. D. Liao M. Walther T. C. (2020). Structure and catalytic mechanism of a human triacylglycerol-synthesis enzyme. *Nature* 581 323–328. 10.1038/s41586-020-2289-6 32433611 PMC7398557

[B167] Sukenik A. Carmeli Y. (1990). Lipid synthesis and fatty acid composition in *Nannochloropsis* sp. (eustigmatophyceae) grown in a light-dark cycle. *J. Phycol.* 26 463–469. 10.1111/j.0022-3646.1990.00463.x

[B168] Talukder M. M. Sim M. F. M. O’Rahilly S. Edwardson J. M. Rochford J. J. (2015). Seipin oligomers can interact directly with AGPAT2 and lipin 1, physically scaffolding critical regulators of adipogenesis. *Mol. Metab.* 4 199–209. 10.1016/j.molmet.2014.12.013 25737955 PMC4338318

[B169] Tamm L. K. Hong H. Liang B. (2004). Folding and assembly of β-barrel membrane proteins. *Biochim. Biophys. Acta* 1666 250–263. 10.1016/j.bbamem.2004.06.011 15519319

[B170] Tanaka A. De Martino A. Amato A. Montsant A. Mathieu B. Rostaing P. (2015). Ultrastructure and membrane traffic during cell division in the marine pennate diatom *Phaeodactylum tricornutum*. *Protist* 166 506–521. 10.1016/j.protis.2015.07.005 26386358 PMC4710849

[B171] Tauchi-Sato K. Ozeki S. Houjou T. Taguchi R. Fujimoto T. (2002). The surface of lipid droplets is a phospholipid monolayer with a unique fatty acid composition. *J. Biol. Chem.* 277 44507–44512. 10.1074/jbc.M207712200 12221100

[B172] Taurino M. Costantini S. De Domenico S. Stefanelli F. Ruano G. Delgadillo M. O. (2018). SEIPIN proteins mediate lipid droplet biogenesis to promote pollen transmission and reduce seed dormancy. *Plant Physiol.* 176 1531–1546. 10.1104/pp.17.01430 29203558 PMC5813562

[B173] Thiam A. R. Forêt L. (2016). The physics of lipid droplet nucleation, growth and budding. *Biochim. Biophys. Acta* 1861 715–722. 10.1016/j.bbalip.2016.04.018 27131867

[B174] Tsai C.-H. Zienkiewicz K. Amstutz C. L. Brink B. G. Warakanont J. Roston R. (2015). Dynamics of protein and polar lipid recruitment during lipid droplet assembly in *Chlamydomonas reinhardtii*. *Plant J.* 83 650–660. 10.1111/tpj.12917 26096381

[B175] Van Mooy B. A. S. Fredricks H. F. Pedler B. E. Dyhrman S. T. Karl D. M. Koblížek M. (2009). Phytoplankton in the ocean use non-phosphorus lipids in response to phosphorus scarcity. *Nature* 458 69–72. 10.1038/nature07659 19182781

[B176] Vancaester E. Depuydt T. Osuna-Cruz C. M. Vandepoele K. (2020). Comprehensive and functional analysis of horizontal gene transfer events in diatoms. *Mol. Biol. Evol.* 37 3243–3257. 10.1093/molbev/msaa182 32918458

[B177] Vieler A. Brubaker S. B. Vick B. Benning C. (2012a). A lipid droplet protein of *Nannochloropsis* with functions partially analogous to plant oleosins. *Plant Physiol.* 158 1562–1569. 10.1104/pp.111.193029 22307965 PMC3320170

[B178] Vieler A. Wu G. Tsai C.-H. Bullard B. Cornish A. J. Harvey C. (2012b). Genome, functional gene annotation, and nuclear transformation of the heterokont oleaginous alga *Nannochloropsis oceanica* CCMP1779. *PLoS Genet.* 8:e1003064. 10.1371/journal.pgen.1003064 23166516 PMC3499364

[B179] Villanova V. Fortunato A. E. Singh D. Bo D. D. Conte M. Obata T. (2017). Investigating mixotrophic metabolism in the model diatom *Phaeodactylum tricornutum*. *Phil. Trans. R. Soc. Lond. B* 372 20160404. 10.1098/rstb.2016.0404 28717014 PMC5516113

[B180] Walther T. C. Chung J. Farese R. V. (2017). Lipid droplet biogenesis. *Annu. Rev. Cell Dev. Biol.* 33 491–510. 10.1146/annurev-cellbio-100616-060608 28793795 PMC6986389

[B181] Wang C.-W. Miao Y.-H. Chang Y.-S. (2014). Control of lipid droplet size in budding yeast requires the collaboration between Fld1 and Ldb16. *J. Cell Sci.* 127 1214–1228. 10.1242/jcs.137737 24434579

[B182] Wang D. Ning K. Li J. Hu J. Han D. Wang H. (2014). *Nannochloropsis* genomes reveal evolution of microalgal oleaginous traits. *PLoS Genet.* 10:e1004094. 10.1371/journal.pgen.1004094 24415958 PMC3886936

[B183] Wang H. Becuwe M. Housden B. E. Chitraju C. Porras A. J. Graham M. M. (2016). Seipin is required for converting nascent to mature lipid droplets. *eLife* 5:e16582. 10.7554/eLife.16582 27564575 PMC5035145

[B184] Wang S. Idrissi F.-Z. Hermansson M. Grippa A. Ejsing C. S. Carvalho P. (2018). Seipin and the membrane-shaping protein Pex30 cooperate in organelle budding from the endoplasmic reticulum. *Nat. Commun.* 9:2939. 10.1038/s41467-018-05278-2 30054465 PMC6063905

[B185] Wang X. Hao T.-B. Balamurugan S. Yang W.-D. Liu J.-S. Dong H.-P. (2017). A lipid droplet-associated protein involved in lipid droplet biogenesis and triacylglycerol accumulation in the oleaginous microalga *Phaeodactylum tricornutum*. *Algal Res.* 26 215–224. 10.1016/j.algal.2017.07.028

[B186] Ward C. Martinez-Lopez N. Otten E. G. Carroll B. Maetzel D. Singh R. (2016). Autophagy, lipophagy and lysosomal lipid storage disorders. *Biochim. Biophys. Acta* 1861 269–284. 10.1016/j.bbalip.2016.01.006 26778751

[B187] Welte M. A. (2015). Expanding roles for lipid droplets. *Curr. Biol.* 25 R470–R481. 10.1016/j.cub.2015.04.004 26035793 PMC4452895

[B188] Weng L.-C. Pasaribu B.-Ping Lin I. Tsai C.-H. Chen C.-S. Jiang P.-L. (2015). Nitrogen deprivation induces lipid droplet accumulation and alters fatty acid metabolism in symbiotic dinoflagellates isolated from *Aiptasia pulchella*. *Sci. Rep.* 4:5777. 10.1038/srep05777 25047647 PMC4105741

[B189] Wimley W. C. (2009). Toward genomic identification of β-barrel membrane proteins: composition and architecture of known structures. *Protein Sci.* 11 301–312. 10.1110/ps.29402 11790840 PMC2373429

[B190] Xin Y. Lu Y. Lee Y.-Y. Wei L. Jia J. Wang Q. (2017). Producing designer oils in industrial microalgae by rational modulation of co-evolving Type-2 Diacylglycerol Acyltransferases. *Mol. Plant* 10 1523–1539. 10.1016/j.molp.2017.10.011 29107033

[B191] Xu Y. Caldo K. M. P. Falarz L. Jayawardhane K. Chen G. (2020). Kinetic improvement of an algal diacylglycerol acyltransferase 1 via fusion with an acyl-CoA binding protein. *Plant J.* 102 856–871. 10.1111/tpj.14708 31991039

[B192] Xu Y. Caldo K. M. P. Pal-Nath D. Ozga J. Lemieux M. J. Weselake R. J. (2018). Properties and biotechnological applications of Acyl-CoA:diacylglycerol Acyltransferase and Phospholipid:diacylglycerol Acyltransferase from terrestrial plants and microalgae. *Lipids* 53 663–688. 10.1002/lipd.12081 30252128

[B193] Yan R. Qian H. Lukmantara I. Gao M. Du X. Yan N. (2018). Human SEIPIN binds anionic phospholipids. *Dev. Cell* 47 248–256.e4. 10.1016/j.devcel.2018.09.010 30293840

[B194] Yang Z.-K. Niu Y.-F. Ma Y.-H. Xue J. Zhang M.-H. Yang W.-D. (2013). Molecular and cellular mechanisms of neutral lipid accumulation in diatom following nitrogen deprivation. *Biotechnol. Biofuels* 6:67. 10.1186/1754-6834-6-67 23642220 PMC3662598

[B195] Yim W. W.-Y. Mizushima N. (2020). Lysosome biology in autophagy. *Cell Discov.* 6:6. 10.1038/s41421-020-0141-7 32047650 PMC7010707

[B196] Yoneda K. Yoshida M. Suzuki I. Watanabe M. M. (2016). Identification of a major lipid droplet protein in a marine diatom *Phaeodactylum tricornutum*. *Plant Cell Physiol.* 57 397–406. 10.1093/pcp/pcv204 26738549

[B197] Yoon K. Han D. Li Y. Sommerfeld M. Hu Q. (2012). Phospholipid:diacylglycerol acyltransferase is a multifunctional enzyme involved in membrane lipid turnover and degradation while synthesizing triacylglycerol in the unicellular green microalga *Chlamydomonas reinhardtii*. *Plant Cell* 24 3708–3724. 10.1105/tpc.112.100701 23012436 PMC3480297

[B198] Zhang Y. Pan Y. Ding W. Hu H. Liu J. (2020). Lipid production is more than doubled by manipulating a diacylglycerol acyltransferase in algae. *GCB Bioenergy* 13 185–200. 10.1111/gcbb.12771

[B199] Zienkiewicz A. Zienkiewicz K. Poliner E. Pulman J. A. Du Z.-Y. Stefano G. (2020). The microalga *Nannochloropsis* during transition from quiescence to autotrophy in response to nitrogen availability. *Plant Physiol.* 182 819–839. 10.1104/pp.19.00854 31740503 PMC6997683

[B200] Zienkiewicz K. Zienkiewicz A. Poliner E. Du Z.-Y. Vollheyde K. Herrfurth C. (2017). *Nannochloropsis*, a rich source of diacylglycerol acyltransferases for engineering of triacylglycerol content in different hosts. *Biotechnol. Biofuels* 10:8. 10.1186/s13068-016-0686-8 28070221 PMC5210179

[B201] Zulu N. N. Zienkiewicz K. Vollheyde K. Feussner I. (2018). Current trends to comprehend lipid metabolism in diatoms. *Prog. Lipid Res.* 70 1–16. 10.1016/j.plipres.2018.03.001 29524459

